# Recurrent latent dynamics in spinal circuits enable a digital twin of autonomic state transitions

**DOI:** 10.1016/j.isci.2026.117971

**Published:** 2026-10-26

**Authors:** Minhye Choo, Mingeun Cho, Jiho Lee, Changhwa Oh, Sungjun Hong, Chaewon Heo, Young-Min Shon, Sungmin Park

**Affiliations:** 1 Department of Convergence IT Engineering, Pohang University of Science and Technology (POSTECH), Pohang, Republic of Korea; 2 Department of Mechanical Engineering, Pohang University of Science and Technology (POSTECH), Pohang, Republic of Korea; 3 Biomedical Engineering Research Center, Samsung Medical Center, Seoul, Republic of Korea; 4 Department of Electrical Engineering, Pohang University of Science and Technology, Pohang, Republic of Korea; 5 Major of Medical Science and Engineering, Pohang University of Science and Technology, Pohang, Republic of Korea

**Keywords:** spinal cord circuits, autonomic regulation, neural population dynamics, recurrent neural network, neural manifold, blood pressure

## Abstract

Physiological signals underlying homeostasis, such as blood pressure, are dynamic and nonlinear, shaped by interactions among diverse neurons. In the spinal intermediolateral nucleus (IML), neurons show heterogeneous, monotonic firing changes during blood pressure transitions, intrinsic to control yet difficult to decode. We introduce a manifold-based model reconstructing internal state dynamics from IML activity, beyond prior descriptive frameworks. Brief spinal stimulation lowering blood pressure showed that single-neuron responses could not explain the nonlinear trajectory, so we derived a low-dimensional circular manifold capturing its cyclical evolution. Embedding the manifold as a dynamic reservoir in a recurrent neural network reconstructed trial-to-trial variability and nonlinear blood pressure dynamics generated by the manifold, without a readout. Our results establish the neural manifold as a generative computational substrate for autonomic physiology, a capability that remains largely unexplored in spinal circuits, and a step toward closed-loop spinal neuromodulation.

## Highlights

• Spinal IML population activity forms a circular manifold of autonomic state
• Latent manifold dynamics encode blood pressure depression and recovery phases
• Autonomic state information emerges from structured population interactions
• A recurrent model embeds spinal manifold dynamics to reproduce neural and BP states

## Graphical abstract

**Figure:**
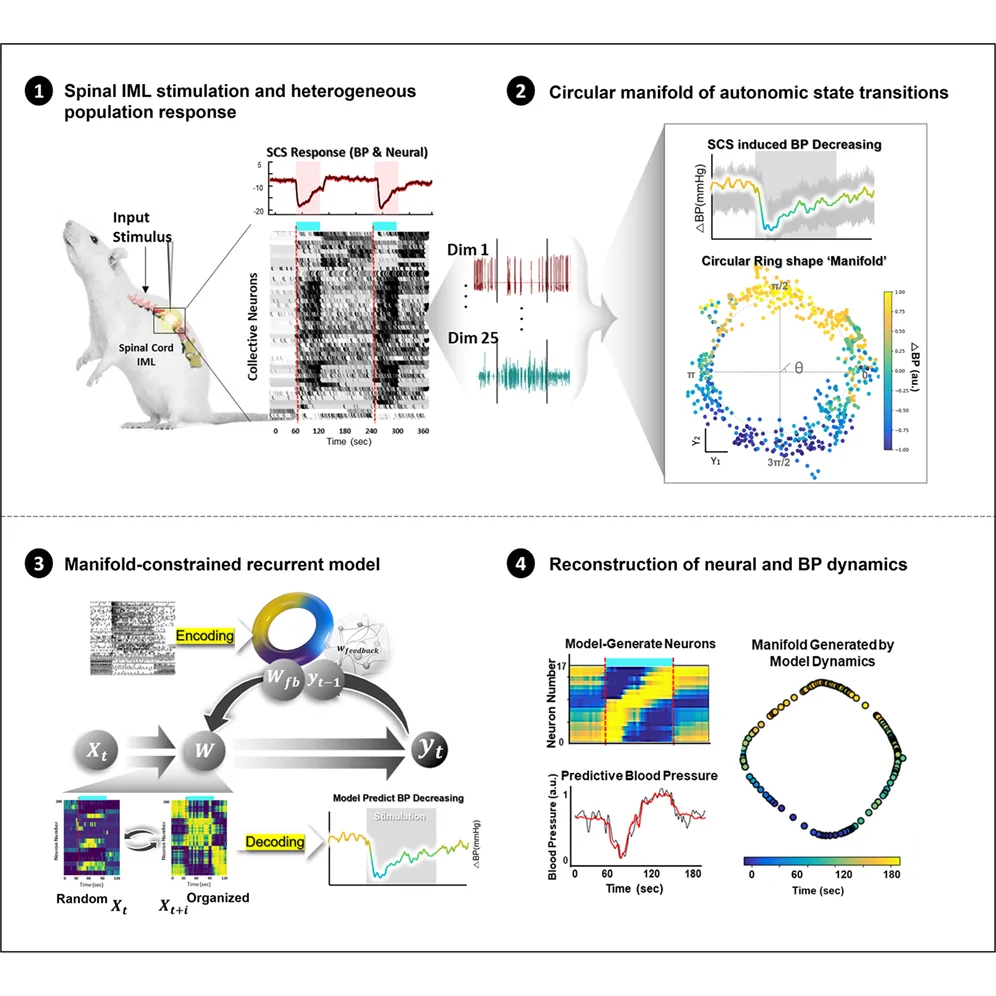

## Teaser

Neuroscience; Computational bioinformatics

## Introduction

Physiological responses emerge from coordinated activity within neural circuits and result from regulated outputs shaped by mutual inhibition and excitation among distinct neuronal types.^1^^,^^2^^,^^3^^,^^4^ Within a single circuit, excitatory, inhibitory, and output neurons coexist, each exhibiting distinct responses to identical external stimuli—some are activated, others suppressed, and some relay output signals to peripheral targets.^5^^,^^6^ These heterogeneous responses are dynamically coordinated through recurrent interactions, forming mechanisms that prevent excessive activity and maintain balance.

The recurrent architecture of neural circuits is fundamental to homeostatic regulation.^7^ Rather than simple on-off switching, homeostasis arises from a balance achieved through ongoing mutual adjustment between excitation and inhibition.^7^^,^^8^^,^^9^ During transient excitation, inhibitory feedback constrains overall output within a functional range.^10^ Such recurrent feedback enables continuous, finely tuned control of physiological variables, including blood pressure (BP), body temperature, and respiration.^11^^,^^12^^,^^13^ Circuits regulating these variables comprise multiple neuron types with specialized roles, allowing each circuit to sustain an internal balance of neural activity.

To decode physiological information—specifically that which mediates homeostatic regulation—from neural responses, it is essential to move beyond single-neuron firing or simple averaging across multiple neurons,^14^ as these approaches can obscure competitive dynamics among heterogeneous neural populations.^15^^,^^16^^,^^17^ Instead, understanding circuit-level interactions through population dynamics is necessary for accurately interpreting the underlying regulatory mechanisms.^18^^,^^19^ Recent studies employing latent space representations and network-based analyses have uncovered nonlinear relationships between neural population activity and physiological functions.^16^^,^^20^^,^^21^ These approaches reveal hidden interaction structures and state transition trajectories not apparent from single-neuron analyses.

The autonomic system, in particular, necessitates a circuit-level perspective due to its reliance on antagonistic interactions and recurrent connectivity, reflecting the complexity and nonlinearity of physiological regulation.^22^^,^^23^^,^^24^ Within this system, the intermediolateral nucleus (IML) of the spinal cord serves as a central sympathetic hub, integrating descending brainstem input and peripheral sensory feedback to regulate BP.^25^^,^^26^^,^^27^^,^^28^ The IML contains both excitatory sympathetic preganglionic neurons (SPNs) and GABAergic interneurons, which together form a closed-loop network coordinating neural dynamics for autonomic regulation^29^^,^^30^^,^^31^^,^^32^^,^^33^ (Figure 1). Although the IML is part of the sympathetic output pathway, it harbors a recurrent microcircuit that enables local modulation via inhibitory interactions, facilitating dynamic responses.^30^^,^^34^^,^^35^ This suggests that the population activity within the IML itself encodes autonomic physiological state transitions.

**Figure 1:**
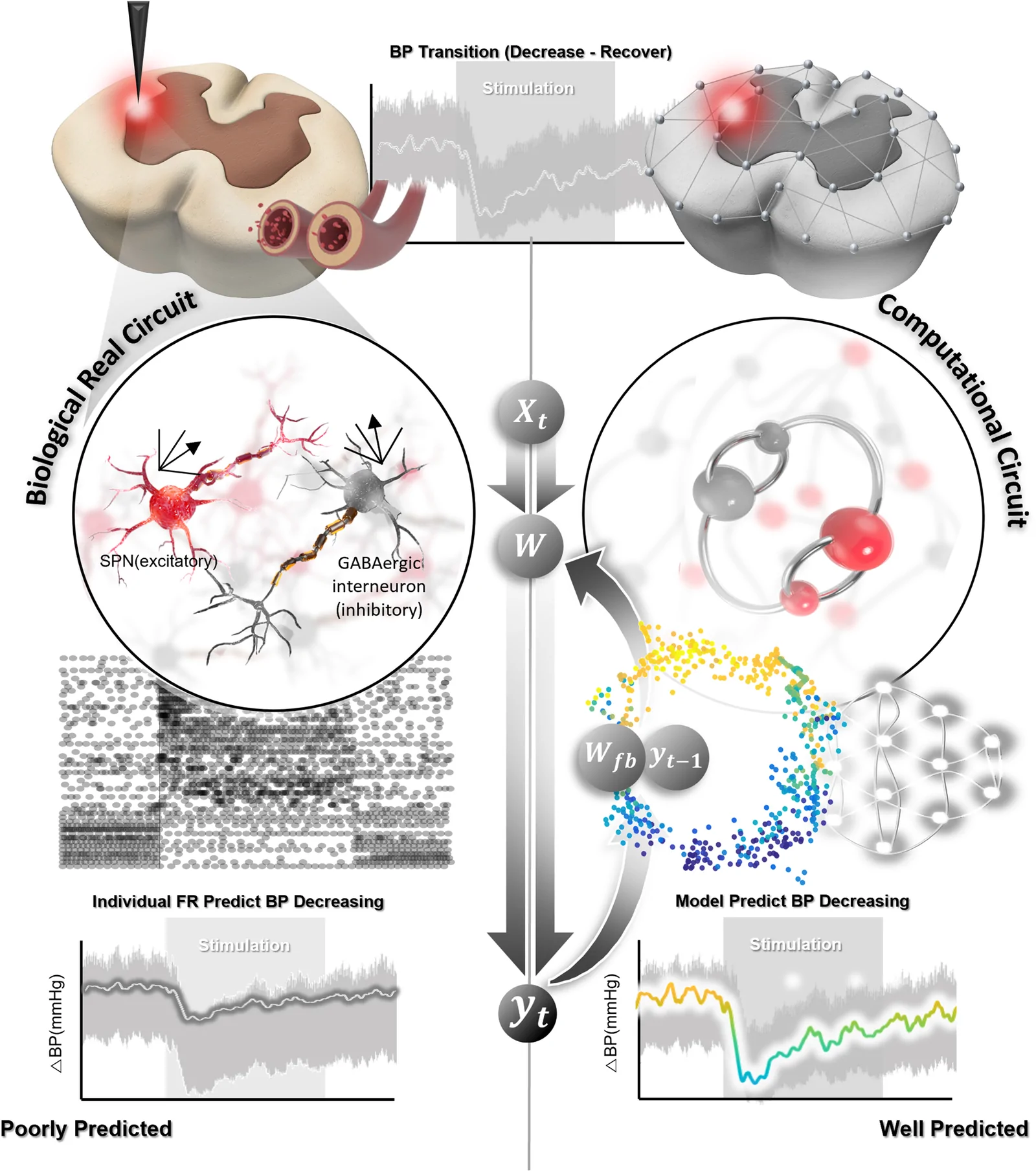
Decoding reflexive transitions: Individual versus modeled circuit dynamics Left (biological circuit): electrical stimulation of the spinal IML elicits transitions in blood pressure (BP) (top). Neurons display heterogeneous and temporally misaligned firing (middle). Linear summation of individual activity (*X*_t_ → *W* → *Y*_t_), without feedback, fails to accurately predict BP dynamics (bottom left). Right (computational model): a recurrent neural network (RNN) simulating IML recurrence embeds the circular manifold as a dynamic reservoir. By integrating current input (*X*_t_) and prior output (*Y*_t−1_) through feedback (*W*_fb_), the model captures time-dependent population dynamics and precisely reconstructs BP transitions (bottom right). Abbreviations are as follows: IML, intermediolateral nucleus; BP, blood pressure.

To investigate how this recurrent architecture mediates physiological transitions, we stimulated the IML while simultaneously recording neural population activity and BP. Consistent with its feedback-driven organization, neuronal responses varied widely: some units increased firing, others decreased, and many remained unchanged.^36^^,^^37^^,^^38^ Despite this heterogeneity, dimensionality reduction revealed a well-organized circular manifold in two-dimensional latent space.^14^^,^^16^^,^^39^^,^^40^^,^^41^^,^^42^ The two extracted orthogonal axes corresponded to the decrease and recovery phases of the BP reflex, reflecting both the temporal structure of the transition and the antagonistic circuit dynamics underlying it. Thus, the manifold not only compresses neural activity into a low-dimensional form but also reflects circuit-level coordination among diverse neurons.^43^^,^^44^ Low-dimensional manifold decoding of stimulus-evoked neural states is not a new approach.^45^^,^^46^^,^^47^ Deriving manifolds from high-dimensional neural signals, such as electroencephalogram (EEG), and mapping them onto behavior has become a common, descriptive framework in neuroscience research. Here, we went a step further and asked whether this manifold could instead serve a generative role. To probe this, we embedded the manifold as a fixed dynamic reservoir within a recurrent neural network.^48^^,^^49^ Rather than directly predicting BP, the manifold updated internal neural states over time, which then generated accurate BP predictions and simultaneously reconstructed underlying population-level dynamics.^50^^,^^51^^,^^52^^,^^53^^,^^54^ Collectively, the IML reservoir model elucidates fundamental computational principles by which a recurrent autonomic circuit governs physiological state transitions, providing a digital twin representation of the spinal IML.

In summary, by modeling an autonomic reflex within a biologically grounded recurrent circuit, we present a computational framework revealing how internal neural structure governs transitions in homeostatic state (Figure 1). Unlike conventional decoding approaches that often neglect intrinsic recurrent dynamics, our model demonstrates how structured population activity drives autonomic transitions. By formalizing internal dynamics as a digital twin of the spinal circuit, the model offers a quantifiable framework for interpreting physiological responses arising from complex neuronal interactions. This approach establishes a foundation for stimulation-based bioelectronic interventions in anatomically complex neural hubs where diverse neuronal populations converge.

## Results

Autonomic regulation preserves systemic homeostasis by dynamically integrating cardiovascular, respiratory, and visceral functions across multiple organ systems. Transitions in autonomic states—manifested as shifts in vascular resistance or cardiac output—are not solely governed by descending brain commands but also depend on intrinsic computations within spinal circuits.^55^^,^^56^^,^^57^ These local computations enable spinal circuits to integrate afferent feedback, maintain autonomous control, and flexibly coordinate organ-specific responses under changing physiological conditions. For BP regulation, corrective responses are initiated by brainstem nuclei but ultimately executed at the spinal level. Specifically, the IML integrates descending autonomic inputs and modulates sympathetic tone via local interactions between excitatory SPNs and GABAergic interneurons. These interactions form a recurrent microcircuit, where GABAergic inhibition—mediated by AT_2_R-dependent activation of the NO/NOS pathway—suppresses SPN excitability, thereby constraining excessive output and maintaining internal stability. Consequently, external stimulation of the IML engages antagonistic local dynamics, resulting in nonlinear and state-dependent changes in sympathetic activity. To examine this, we applied focal electrical stimulation to the IML while simultaneously recording multi-unit neuronal activity and BP. This approach systematically probed how heterogeneous neuronal responses contribute to coherent physiological regulation, enabling us to uncover the internal organization of spinal autonomic computation during externally driven state transitions.

### Spinal stimulation drives coherent autonomic output via heterogeneous neuronal responses

The IML in rats, as in humans, extends from T2 to L2 and conveys sympathetic output to peripheral targets. To capture rapid hemodynamic responses, we delivered focal electrical stimulation to the T13-L1 segment—a region of the spinal IML regulating vascular tone—in male Sprague-Dawley rats (270–320 g), with simultaneous recordings of blood pressure (BP) and multi-unit neural activity (Figure 2A).

**Figure 2:**
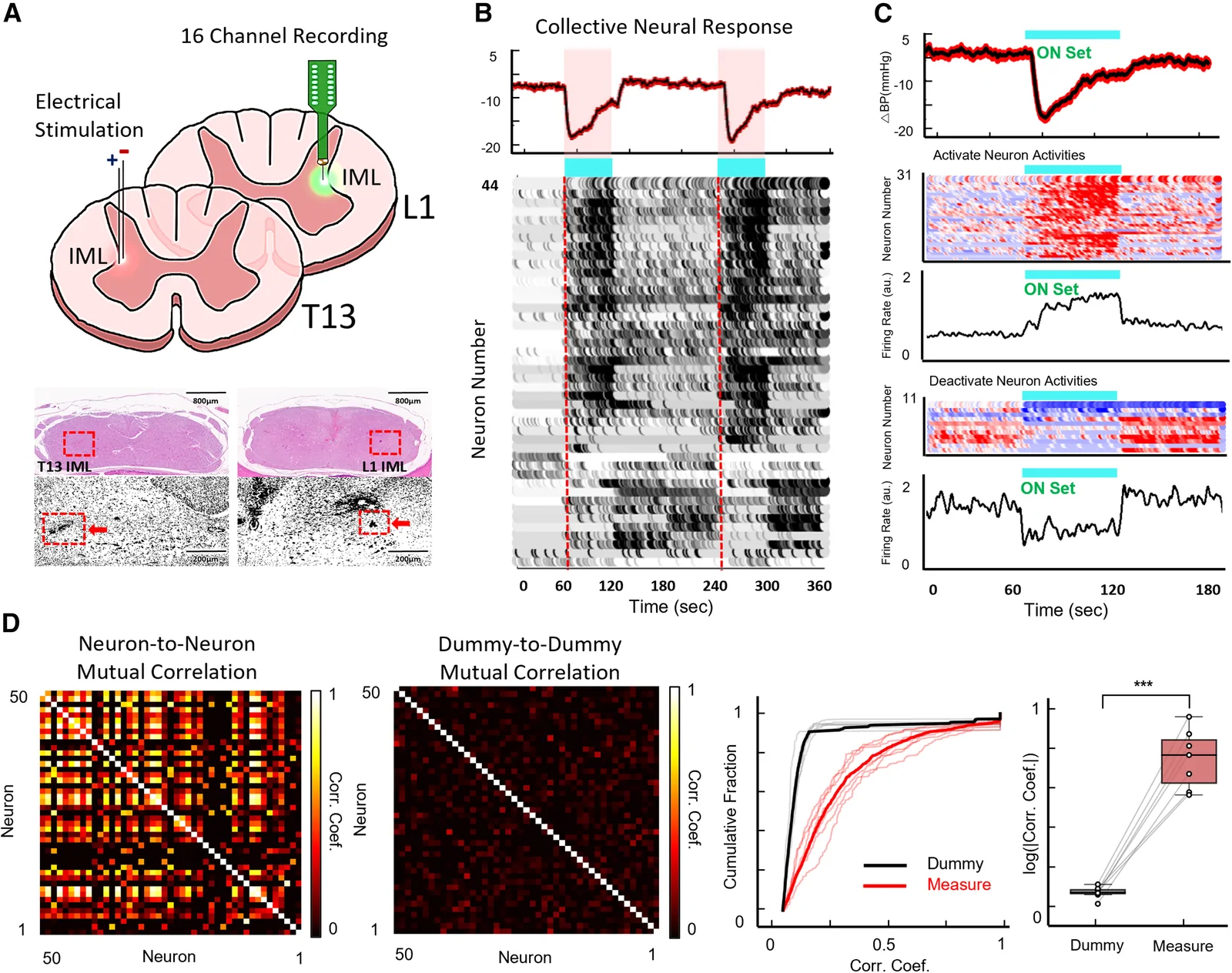
Heterogeneous collective neuronal responses and neuron-to-neuron interactions within the local circuit of the spinal IML (A) Schematic of the experimental setup depicting bipolar electrical stimulation and 16-channel neural recording targeting the T13-L1 region of the IML, with histological validation of electrode placement. (B) Top: representative changes in mean arterial blood pressure (ΔBP) during stimulation. Bottom: population spike firing activity from 44 sorted neurons across pre-, during-, and post-stimulation periods. (C) Top: blood pressure trace (ΔBP) aligned to stimulation onset. Middle: raster plot and peri-stimulus time histogram (PSTH) of neurons with increased firing during stimulation. Bottom: raster plot and PSTH of neurons with decreased firing at stimulation onset. (D) Left: neuron-to-neuron correlation matrix from spike trains during stimulation, revealing structured mutual activity. Middle left: control matrix generated from randomly shuffled spike timings. Middle right: cumulative distribution of correlation coefficients, showing a rightward shift in measured data, with 31.7% of neuron pairs exceeding correlation coefficient = 0.3. Right: boxplot indicating significantly higher correlation among recorded neurons than control (∗∗∗*p* < 0.001).

Each recording session consisted of a 5-min resting baseline followed by a 60-s stimulation period. Spinal stimulation frequencies of 30–40 Hz have been reported to maximize GABAergic interneuron-mediated inhibition of target neurons,^58^ and our previous work similarly found that the BP-lowering effect of IML stimulation was optimized within this 30–40 Hz range.^59^ Based on these findings, we applied 40 Hz stimulation in the present study. In 8 rats, 40 Hz stimulation consistently evoked a transient reduction in BP, tightly time-locked to the stimulation window (Figure 2B, top), demonstrating a reproducible hemodynamic reflex. Concurrently, stimulation induced heterogeneous yet coordinated patterns of neural activity within the IML, indicating that structured population dynamics may underlie the observed physiological transitions.

Despite consistent physiological output, neuronal responses within the IML exhibited pronounced heterogeneity. Spike sorting from 16-channel electrode recordings yielded 30–50 well-isolated neurons per animal. Population raster plots revealed neurons with increased, decreased, or unchanged firing rates in response to stimulation (Figure 2B, bottom; Figure 2C). In a representative case, 31 of 44 sorted units showed increased firing, 11 showed decreased activity, and 2 exhibited no clear modulation (Figure 2C, middle and bottom). Despite identical stimulation, individual IML neurons displayed distinct changes in firing rate, varying in direction, timing, and magnitude. This suggests that BP transitions following spinal cord stimulation arise from coordinated interactions among distinct neuronal populations, rather than uniform spiking activity.

As further evidence of circuit-level organization, we assessed whether this coordination was reflected in structured temporal relationships among neurons. We computed pairwise spike-train correlations during the stimulation window to determine whether activity patterns were random or exhibited structured coactivation. If neurons were independently active, correlation values would approximate zero. Two conditions were compared: one using original spike trains, preserving neuron-to-neuron synchrony, and the other using time-shuffled data that maintained firing rates but disrupted spike timing. In the original condition, the correlation matrix revealed distinct clusters of strongly correlated neuron pairs (Figure 2D, left), whereas the shuffled control showed no such clusters (Figure 2D, middle left). To quantitatively assess matrix structure, cumulative distributions of pairwise correlation coefficients were compared across conditions. In the matrix constructed from neurons with preserved firing patterns, approximately one-third of neuron pairs (mean = 31.7% across animals) exhibited correlation coefficients above 0.3. In contrast, in the matrix based on time-shuffled spike trains—where firing rates were preserved but temporal structure was disrupted—only 5.7% of pairs exceeded this threshold (Figure 2D, middle right and right; ∗∗∗*p* < 0.001, paired *t* test). These findings indicate that, although individual neurons differ in firing pattern and spike timing, their activity is not independent but reflects temporally organized coordination governed by a shared population dynamic. To explore how this shared dynamic evolves over time and encodes physiological state transitions, we next projected the population activity into a low-dimensional latent space (Figure 3A).

**Figure 3:**
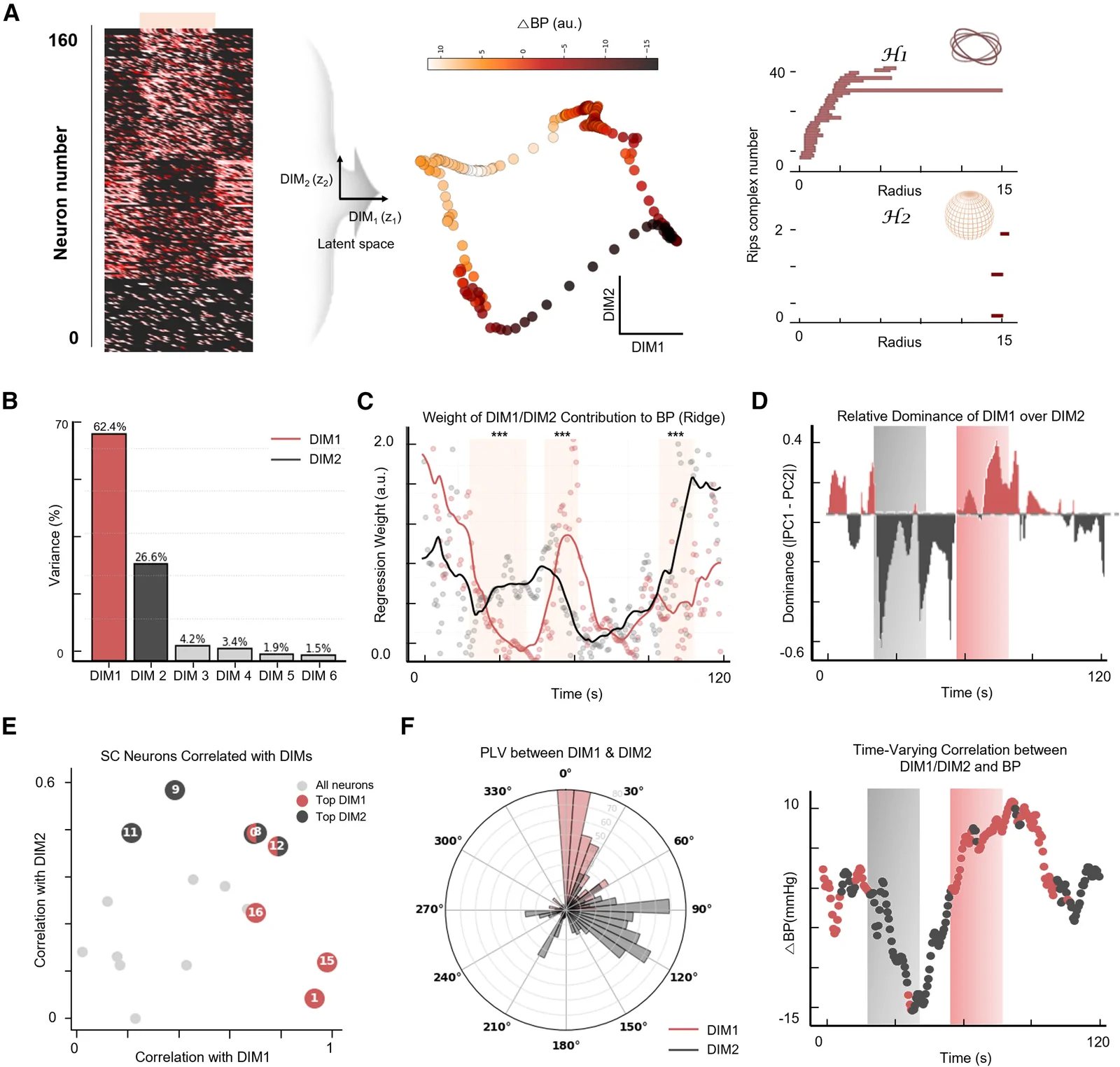
Low-dimensional manifold compresses spinal dynamics underlying stimulus-evoked blood pressure transitions (A) Neural population activity from the IML was projected onto a low-dimensional latent space via nonlinear dimensionality reduction (Isomap). The stimulation-evoked trajectory formed a circular manifold, with trajectory color reflecting blood pressure change (ΔBP). Persistent homology confirmed a nontrivial topological loop (non-zero H_1_), consistent with circular latent dynamics. (B) Variance explained by each Isomap dimension showed that the first two dimensions (Dim1 and Dim2) accounted for 62.4% and 26.6% of the total variance, respectively. (C) Ridge regression assessed the time-varying contributions of Dim1 and Dim2 to blood pressure. Asterisks denote time windows in which the difference between Dim1 and Dim2 regression weights was significant (*p* < 0.001) for at least 5 consecutive seconds, indicating phase-specific recruitment of distinct latent components during physiological transitions. (D) Top: a relative dominance index was calculated by subtracting the contribution of Dim2 from Dim1. Bottom: this index revealed that Dim1 dominated during the falling phase of blood pressure, while Dim2 was dominant during recovery, indicating a temporal shift in latent dynamics. (E) The five neurons most strongly correlated with each latent dimension were identified (top DIM1-aligned, red; top DIM2-aligned, black). Two neurons (indices 8 and 12) ranked among the top five for both dimensions and are marked with bicolor (half red, half black) markers, revealing distinct subgroups preferentially aligned with either Dim1 or Dim2 and supporting functional segregation within the population. (F) Phase-locking analysis of these subgroups demonstrated divergent phase preferences, suggesting that phase-shifted ensemble dynamics underlie encoding of physiological state transitions. Abbreviations are as follows: DIM, dimension.

### Latent projection of spinal activity reveals a circular low-dimensional structure underlying autonomic reflexes

Neural population activity recorded from the IML was projected into a low-dimensional latent space using Isomap, a nonlinear dimensionality reduction method that preserves the geometric relationships of curved manifolds (Figure 3A). Despite the fragmented and temporally disorganized firing of individual neurons, the latent projection revealed a smooth, circular trajectory aligned with the BP transition. This finding suggests the presence of coherent population dynamics embedded within the spinal circuit. To assess the topological significance of the observed circular trajectory, we applied persistent homology. The analysis identified a prominent first homology class (H_1_), indicating a one-loop recurrent structure, while the second homology (H_2_) was negligible. These results support the interpretation that the manifold lacks higher-dimensional voids and can be effectively represented in two dimensions.

To further characterize the manifold’s functional structure, we evaluated the variance captured by each Isomap-derived latent dimension. The first two dimensions (Dim1 and Dim2) accounted for 62.4% and 26.6% of the total variance, respectively—together explaining nearly 90% of the population activity (Figure 3B; Figure S1). This result is consistent with the earlier topological finding that the latent structure is effectively captured within two dimensions.

To confirm that this circular structure reflects the intrinsic geometry of the underlying population activity rather than an artifact of the dimensionality-reduction method used, we compared Isomap against principal component analysis (PCA), locally linear embedding (LLE), and an autoencoder across *n* = 7 trials (Figure S2). Only Isomap preserved a closed, non-self-intersecting circular trajectory (Figure S2A), with the largest and most clearly separated H_1_ persistent-homology feature among the four methods (Figure S2B). Isomap also achieved significantly higher geodesic distance preservation than the other three methods (paired Wilcoxon signed-rank test, *p* < 0.05 for all comparisons), reaching near-maximal preservation with only two latent dimensions, whereas the alternatives did not even with six (Figures S2D and S2E). Using only the first two dimensions of each method to fit BP, Isomap achieved superior reconstruction of the BP trajectory among the four methods (Figures S2C and S2F). Together, these results establish Isomap as the method that most faithfully preserves the geometric and topological structure of IML population activity, motivating a closer examination of how its two latent dimensions jointly encode the BP reflex.

Mathematically, constructing a circular trajectory in latent space requires two phase-offset axes. If the empirically derived manifold reflects such a structure, its constituent dimensions should contribute in a time-dependent and functionally distinct manner. To test this, we applied a sliding-window ridge regression to calculate the time-varying contributions (absolute weights) of Dim1 and Dim2 to BP dynamics. Temporal analysis revealed that their contributions shifted across different phases of the transition. Statistically significant intervals, defined as periods where *p* < 0.001 persisted for more than 7 s, revealed distinct phases of dominance between the two axes (Figure 3C). We further computed a dominance index (Dim1 − Dim2) to quantify these asymmetries. Dim1 exerted greater influence during the BP drop, whereas Dim2 became more prominent during the recovery phase (Figure 3D). These temporal dynamics indicate that the manifold’s axes are not static compressions but instead encode phase-shifted components of the reflexive transition.

To determine whether these axes correspond to distinct neural subgroups, we identified neurons with the highest correlation to each dimension. Specifically, we selected five neurons most strongly aligned with Dim1 and five with Dim2 (Figure 3E). Phase analysis of these neurons, computed by binning the circular trajectory into discrete segments, revealed distinct preferred phases for the two subgroups (Figure 3F). This finding implies that the two latent dimensions extract phase-specific activity patterns from neural ensembles, each reflecting distinct aspects of the autonomic reflex. Given the opposing trajectories of Dim1 and Dim2 in contributing to BP dynamics, and the phase separation of the neurons aligned with each axis, we infer that the two dimensions reflect antagonistic neural mechanisms within the IML—specifically, excitatory and inhibitory functional roles. These axes do not simply represent excitatory or inhibitory neuron types; rather, they correspond to dominant neural ensembles that govern different phases of the reflex: one subgroup becomes more active during the pressure drop and the other during recovery. Such phase-shifted dominance supports dynamic circuit stability. The population activity projected onto these complementary axes occasionally departs from balance but consistently returns to an equilibrium point, generating a circular trajectory. As a result, the phase along this manifold compactly encodes both the temporal evolution of BP and the dynamic balance between opposing neural drives that define autonomic responses.

### Functional encoding of hemodynamics within the latent neural manifold

Although the manifold is constructed from the activity of individual neurons, it remains unclear whether a small subset of neurons—those contributing most strongly to the latent dimensions—could explain the physiological transitions as effectively as the manifold itself. Addressing this issue is central to understanding whether the latent structure is simply a byproduct of neural firing or a functionally meaningful representation of autonomic state. The fact that different neurons contribute differentially to Dim1 and Dim2 does not imply that these neurons individually encode BP dynamics. Instead, we sought to determine whether the manifold embeds intrinsic circuit dynamics—arising from antagonistic neuronal interactions—that are not recoverable by summing over individual responses.

To investigate this, we selected a minimal set of six neurons: three showing strong contributions to Dim1 and three to Dim2, all of which were highly active during stimulation (Figure 4A). These neurons were not arbitrarily chosen but identified as key contributors to the construction of the latent space. Although the sample size was small, their contribution weights spanned both orthogonal axes of the manifold, forming a balanced basis for trajectory generation. Using only these six neurons, we reconstructed a circular latent trajectory closely resembling the full manifold derived from the entire population (Figure 4B). Phase values derived from this trajectory provided a compact, time-evolving representation of neural state, maintaining continuous alignment with both stimulation timing and BP transitions.

**Figure 4:**
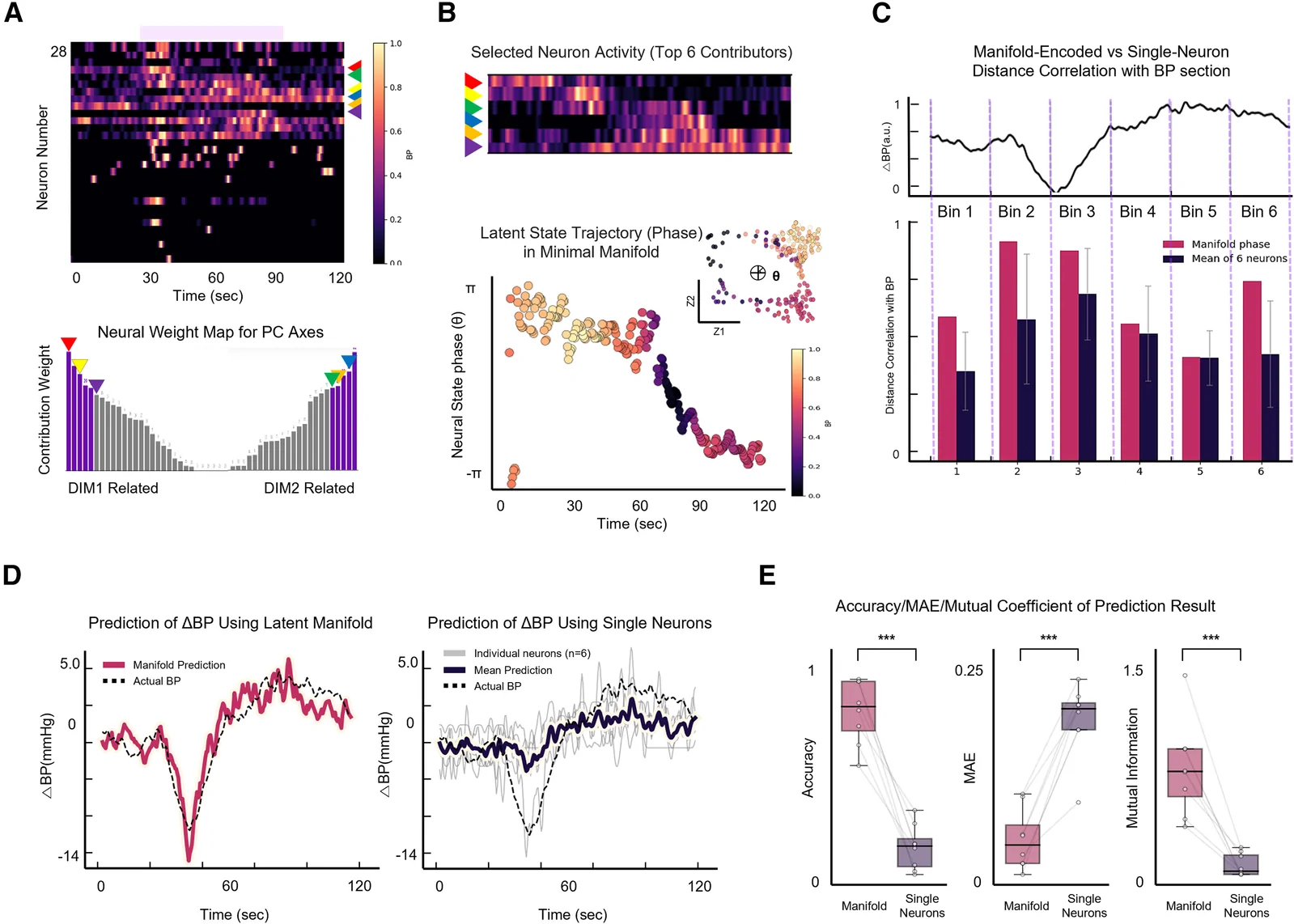
Manifold-based neural encoding outperforms individual neurons in predicting physiological state transitions (A) Among neurons with stimulus-locked responses, the top six were selected based on high firing rates and strong contributions to the Isomap dimensions (Dim1 and Dim2). Raster plots (top) display the temporal firing patterns of each neuron; the bar plot (bottom) quantifies each neuron’s contribution to both dimensions. (B) A minimal circular manifold was reconstructed from these six neurons, and the latent trajectory was used to compute neural phase, which was temporally aligned with blood pressure fluctuations. (C) Blood pressure (ΔBP) was segmented into six time bins, and distance correlation was calculated between ΔBP and either manifold-derived phase (magenta) or the mean activity of the six neurons (gray). The manifold-based representation consistently yielded higher correlation values, particularly during dynamic BP transitions (bins 2, 3, and 6). (D) Linear regression predicted ΔBP using either manifold phase (red) or firing rates from the six neurons (purple). The manifold-based prediction more closely tracked BP fluctuations, whereas single-neuron-based prediction exhibited higher trial-to-trial variability and poorer alignment with actual BP dynamics. (E) Across animals (*n* = 7), manifold-based decoding significantly outperformed single-neuron models in prediction accuracy (R^2^), mean absolute error (MAE), and mutual information (MI). ∗∗∗*p* < 0.001, paired *t* test.

While the manifold trajectory visually aligned with BP transitions, we performed a quantitative comparison to determine whether the manifold phase encoded more physiological information than the firing activity of the individual neurons from which it was derived. To this end, we conducted a distance correlation analysis between ΔBP and either the manifold-derived phase or the average activity of the six contributing neurons (Figure 4C). The manifold phase consistently showed stronger correlations with ΔBP, particularly during transition phases (bins 2, 3, and 6), indicating that the trajectory captures temporally structured dynamics that are diluted or lost at the level of individual neurons. Thus, the manifold does not merely represent a compression of neural activity but reveals a circuit-level organization that aligns with physiological transitions.

To further assess whether this latent representation enhances predictive power, we trained linear regression models to reconstruct ΔBP using either the manifold phase or the six neurons’ activity (Figure 4D). The manifold-based model accurately tracked BP fluctuations and reproduced trial-to-trial dynamics, whereas the single-neuron models demonstrated reduced fidelity and higher variability. Group-level analyses across eight animals confirmed that manifold-based decoding significantly outperformed neuron-based models in multiple metrics, including R^2^, mean absolute error (MAE), and mutual information (MI) (Figure 4E; ∗∗∗*p* < 0.001, paired *t* test; Figure S3).

These findings indicate that the essential information underlying hemodynamic transitions is not embedded in the firing patterns of individual neurons but emerges through their structured interactions. The latent manifold encoding these intrinsic dynamics carries richer physiological information and reflects a circuit-level representation that surpasses what can be inferred from the activity of any subset of neurons (Figure S4). The presence of consistent manifolds across animals suggests that these intrinsic dynamics form a shared representational structure—one that can be geometrically aligned to enable a unified frame of neural interpretation.

### Geometric alignment enables a unified neural representation of hemodynamic responses

Circular neural manifolds consistently emerged across all seven animals following stimulation (Figure 5A), but the constructed axes within each trajectory differed in their relative weighting, making them switchable between the two opposing phases of depression and recovery. This variability likely arose from differences in sampled neural populations, electrode placement, or relative contributions to the latent space. Consequently, in some animals, one dimension (e.g., Dim1) predominantly reflected BP depression, while in others it captured the recovery phase. Additional inter-subject differences extended beyond axis interpretation to the geometry of the circular trajectories themselves. Even when the overall shape remained circular, each animal exhibited a distinct ring with unique orientation, radius, and angular progression (Figures 5A and 5B), resulting in geometrically distinguishable manifolds. These idiosyncrasies limit the interpretability of neural trajectories without external references such as stimulation onset or ΔBP measurements. Thus, even when each manifold encodes a similar sequence of physiological transitions, their native coordinate frames are not directly comparable across animals. To enable cross-subject interpretation of circuit-level responses, it is necessary to realign each manifold into a shared reference frame defined by consistent physiological transitions—specifically, the trajectory from ΔBP depression to recovery.

**Figure 5:**
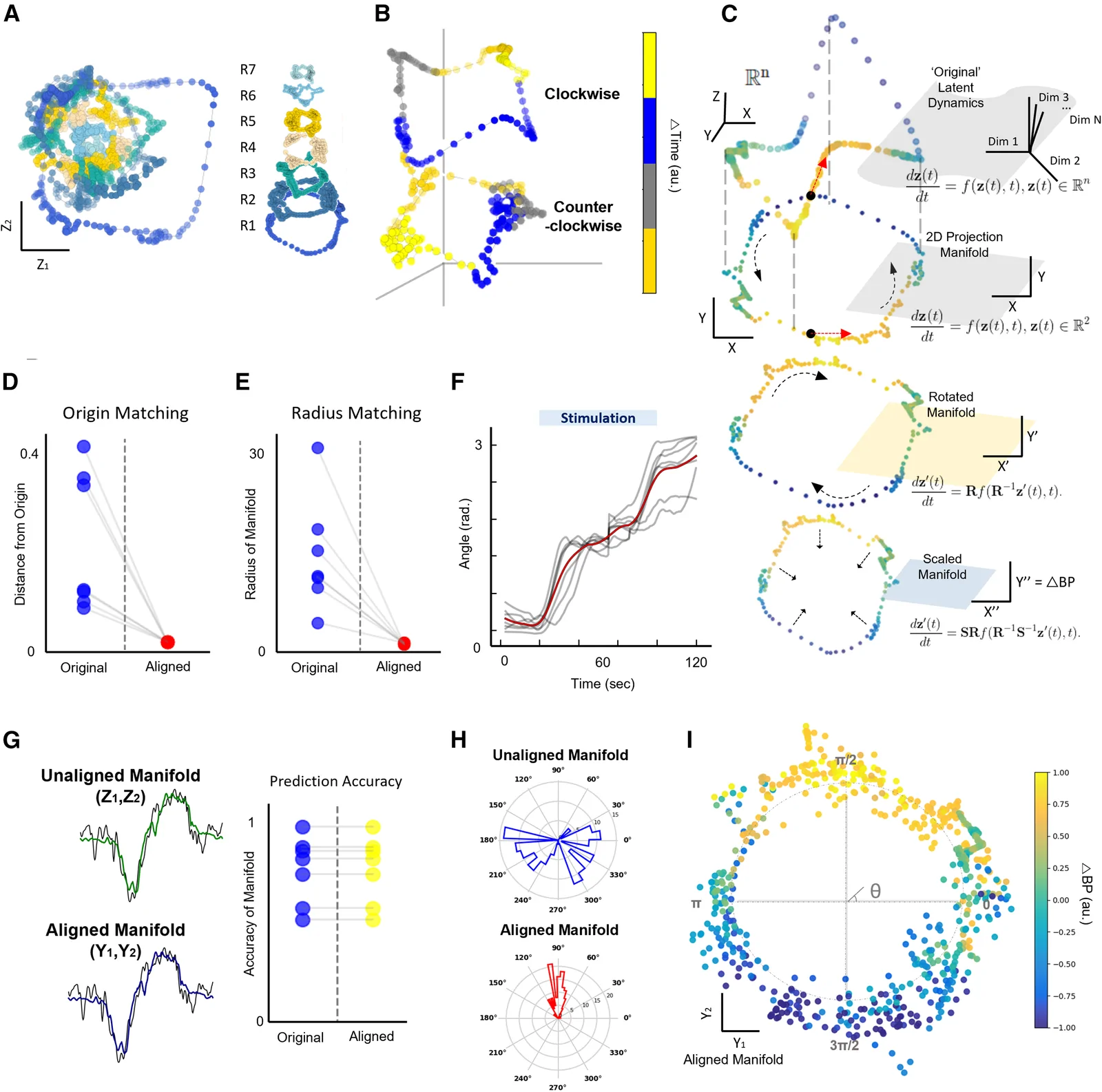
Alignment of individual neural manifolds reveals conserved population structure underlying hemodynamic dynamics (A) Manifold trajectories from individual animals (*n* = 7) exhibited similar circular patterns but differed in size, orientation, and position. (B) The direction of time progression across manifolds varied, with both clockwise and counterclockwise trajectories observed. (C) Linear transformations (translation, rotation, and scaling) aligned individual manifolds into a common reference frame, revealing a shared latent structure across animals. Blood pressure values (color-coded ΔBP) remained continuously organized along the transformed trajectory. (D and E) Individual manifolds were aligned by translating their centers to the origin (D) and scaling to fit within a unit circle (E), standardizing position and radial extent across subjects. (F) Angular trajectories were standardized into a common direction following stimulation, unifying the rotational orientation of the manifold. (G) Prediction accuracy of blood pressure using second-order regression was comparable between unaligned and aligned manifolds, indicating minimal effect of alignment on decoding performance. (H) During the ΔBP depression range, corresponding angular phases in the aligned manifolds became more consistent across animals. (I) Overlay of four aligned manifolds produced a unified circular structure, reprojected along a new vertical axis representing blood pressure magnitude.

To achieve this, we applied a series of linear transformations—including translation, rotation, and scaling—to align the subject-specific manifolds into a standardized coordinate system (Figure 5C). The centers of each trajectory were translated to the origin, radii were normalized to a unit circle, and angular direction was standardized across animals. Critically, the vertical axis of each manifold was reoriented to reflect the peak ΔBP value, providing a physiologically meaningful frame axis (*y* = ΔBP). Following this alignment, geometric variability across subjects was markedly reduced. The manifold centers and radii converged to consistent values (Figures 5D and 5E), and angular trajectories became unidirectional across all animals (Figure 5F).

This normalization process preserved the physiological information embedded in each trajectory. BP decoding using second-order regression showed no loss of accuracy following alignment (Figure 5G). Moreover, the angular phase associated with ΔBP depression became more consistent across animals (Figure 5H), indicating that the aligned trajectories captured a shared temporal structure. When all aligned trajectories were overlaid, the composite manifold exhibited a coherent circular form, with ΔBP magnitude reliably mapped onto a common vertical axis (Figure 5I).

By reducing inter-subject geometric variability and aligning trajectories based on shared physiological transitions, each manifold was stripped of subject-specific idiosyncrasies and transformed into a function-centered representation. Aligned manifolds no longer reflect individual sampling biases or measurement differences but instead encode the core dynamics of the autonomic state transition—specifically, the depression and recovery of BP. As a result, models trained to decode hemodynamic transitions from the manifold can now operate on a shared phase structure across animals, ensuring that the inferred trajectories reflect consistent underlying dynamics rather than animal-specific geometry. The aligned manifold provides a generalizable basis for physiological decoding, as well as a stable scaffold for biologically grounded modeling.

### Neural dynamic modeling captures structured population activity and hemodynamic responses

Having established a stable, shared latent structure that encapsulates the core dynamics of stimulus-driven neural and hemodynamic responses, we next evaluated its functional validity. Specifically, we tested whether this recurrent trajectory alone could reliably capture the internal organization of IML circuit dynamics and enable both the reconstruction of neural activity and the simulation of state transitions.

To determine whether the identified manifold structure supports generative modeling of recurrent circuit dynamics, we developed a recurrent neural network termed the IML computational model. This architecture incorporates the manifold as both a latent representation and a dynamic reservoir, capturing the intrinsic recurrence of the spinal IML. Built on first-order reduced and controlled error (FORCE) learning (Figures 6A and 6B), the IML model generates internal neural states and BP outputs with minimal external input.

**Figure 6:**
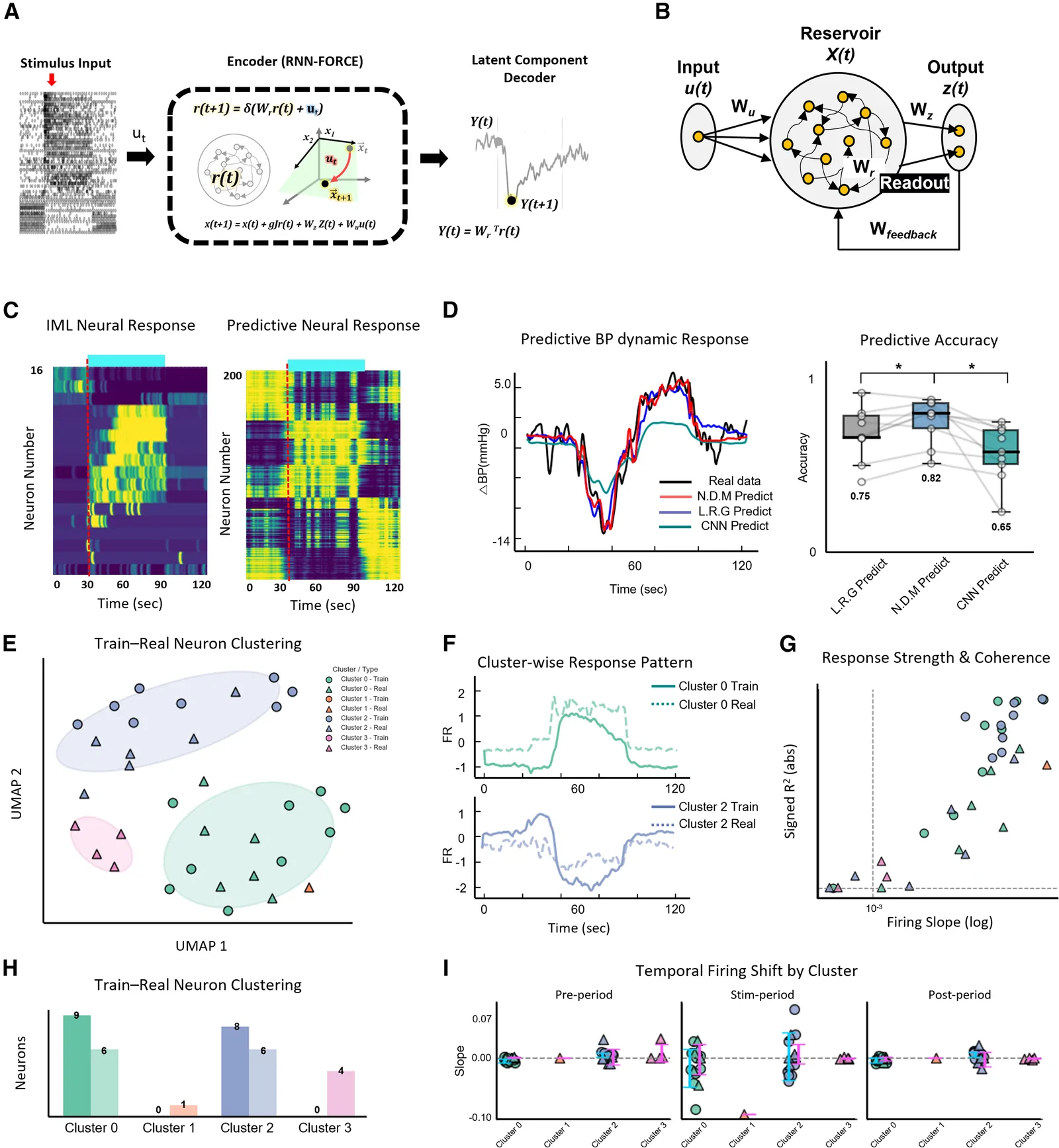
Manifold-based recurrent neural dynamics for predicting hemodynamic states and neural responses (A) Model architecture. The stimulus input *u*(*t*) (binary signal indicating stimulation onset) is integrated with the previous latent state r(t), updating the reservoir state via a recurrent encoder. The reservoir state evolves as *r*(*t*+1) = δ(*W*_*r*_*r*(*t*)+*W*_*u*_*u*(*t*)), and the predicted latent output is decoded as $y=W_{r}^{T}x$ to estimate the next blood pressure value and latent manifold position. (B) Internal encoder structure based on FORCE learning. The input *u*(*t*) and feedback *z*(*t*-1) jointly drive a recurrent reservoir *x*(*t*), whose internal state dynamics are shaped by fixed latent state X(t) and feedback connections *W*_*feedback*_, serving as a computational analog of population activity in the IML. (C) Comparison of experimentally observed neural activity (left; 16 sorted IML neurons) and predicted activity from 200 computational units (right) revealed qualitatively similar activation patterns. (D) Left: blood pressure prediction using different models. The neural dynamic model (NDM; blue) outperformed linear regression (LRG; red) and convolutional neural network (CNN; orange) in reconstructing ΔBP dynamics (black). Right: prediction accuracy was significantly higher for NDM (mean = 0.82) than for LRG (mean = 0.75) or CNN (mean = 0.65) (∗*p* < 0.05). (E) UMAP projection of neural units based on response features revealed four distinct clusters. Two major clusters (clusters 0 and 2) included both model-generated (circles) and real neurons (triangles) with balanced distribution. (F) Averaged firing rate traces from the two major clusters showed consistent temporal patterns between real (dashed) and predicted (solid) neurons. Top: stimulus-induced activation cluster (cluster 0). Bottom: stimulus-induced suppression cluster (cluster 2). (G) Cluster-level comparison of firing slope and linear fit during stimulation. Clusters 0 and 2 showed strong, coherent responses, while minor clusters were distributed in regions with lower slope and fit. (H) Proportion of real and predicted neurons in each cluster. Real/predicted distribution was balanced in clusters 0 (54%/46%) and 2 (47%/53%), indicating consistent response structure. (I) Temporal slope of firing rate in each cluster across pre-, stimulation, and post-periods. Both real and model-derived neurons exhibited similar shift patterns, with significant modulation during stimulation. (Cluster 1 comprised a single neuron whose response amplitude was substantially larger than that of cluster 0 despite a highly similar temporal response shape [*Z* score-normalized correlation, r = 0.978], and is therefore interpreted as an amplitude-driven outlier rather than a distinct functional subtype).

In the IML model, stimulus-timed input signals are delivered to a recurrent reservoir composed of densely interconnected units. The architecture was intentionally designed to emulate the anatomical recurrence of the spinal IML, where intrinsic feedback among neurons governs reflexive transitions. The internal state of the reservoir is updated at each time step through recurrent feedback and weighted input integration, reflecting this circuit-level organization. The manifold structure is implicitly embedded into the recurrent reservoir via FORCE-based training, enabling the network to simulate population-level neural dynamics consistent with the latent geometry. Neural units within the reservoir update their activity according to manifold-constrained dynamics, forming population-wide firing patterns that evolve over time. A comparison of predicted and actual neural firing patterns revealed strong temporal correspondence (Figure 6C; Figure S5), supporting the model’s capacity to reproduce biologically grounded dynamics.

The population firing pattern, accumulated through the updating of neural units within the model, serves as an internal substrate shaped during training. The final output decoded by the model is the predicted BP evoked in response to stimulation. When evaluated on BP prediction, the IML model outperformed both linear regression and convolutional neural network baselines (Figure 6D), achieving the highest decoding accuracy (0.82) with statistical significance (*p* < 0.05). These results demonstrate that a biologically inspired recurrent structure constrained by manifold geometry more effectively captures stimulus-driven physiological responses than conventional architecture.

To assess the structural consistency between predicted and observed neural patterns, we clustered neurons from both groups based on rich response features. Uniform manifold approximation and projection (UMAP) revealed four distinct clusters, with two major groups containing a balanced mixture of real and predicted neurons (Figures 6E–6H). These clusters exhibited consistent average firing responses (Figure 6F), and coherence analyses confirmed strong, directional activity during stimulation (Figure 6G). Finally, both real and modeled neurons displayed similar temporal firing shifts across stimulus phases (Figure 6I), indicating that the model captures not only aggregate dynamics but also temporally structured neural responses.

Collectively, these findings demonstrate that manifold-based recurrent modeling provides a robust framework for decoding physiological states while preserving biologically interpretable features of spinal circuit dynamics.

## Discussion

The findings in this study demonstrate how recurrent spinal circuits within the IML generate structured neural dynamics that encode autonomic reflexes, constructing a computational manifold model that captures this latent mechanism. Stimulation evoked staggered patterns of excitatory and inhibitory activity across the IML neuronal population. When analyzed collectively, these responses formed a temporally organized antagonistic structure aligned with distinct physiological transitions—specifically, BP depression and recovery. This dynamic was embedded in a circular, low-dimensional manifold, where two orthogonal axes represented functionally opposing neural dynamics that intrinsically structured the reflex—specifically, transient shifts between excitatory-dominant and inhibitory-dominant population activity corresponding to phases of BP depression and recovery, respectively. The manifold thus encapsulates more than a descriptive mapping of physiological change; it embodies an intrinsic feedback logic underlying the organization of spinal autonomic circuits. To validate this interpretation, the manifold structure was embedded into a recurrent neural network model, which reproduced both population-level neural responses and BP dynamics with high fidelity. Two principal insights emerge from these findings: (1) autonomic state transitions are governed not by individual neural responses but by circuit-level antagonistic interactions structured across time, and (2) by embedding antagonistic transitions within a circular trajectory, the manifold establishes a recurrent network model that drives physiological state transitions through structured neural dynamics, even in a digital environment.

Prior studies have shown that stimulation of IML neurons induces local inhibitory feedback through circuit-intrinsic antagonism^60^: chemical activation of IML interneurons has been shown to trigger GABAergic inhibition of SPNs,^13^ and antidromic stimulation of individual SPNs transiently suppresses firing in neighboring SPNs via local feedback.^14^ These findings indicate that even within a nucleus traditionally viewed as an excitatory output center, inhibitory loop architectures are embedded to regulate reflexive dynamics. Our results extend this understanding by demonstrating that antagonistic coordination is temporally structured at the population level. Some neurons responded to stimulation with immediate excitation that gradually declined, while others were initially silent but became active later during recovery. Although these staggered dynamics can be discerned from raster plots when examined neuron by neuron, such representations do not quantify which pattern predominates at each moment. Averaging across neurons further obscures these relationships, as opposing responses can cancel each other out and the resulting signal may lose any interpretable link to physiological function.

In contrast, the manifold constructed from population activity provides a continuous trajectory whose orthogonal axes correspond to the distinct phases of BP transition—depression and recovery. Because each axis reflects a latent feature shaped by temporally structured antagonism, the manifold reveals whether the population activity at a given moment contributes to suppression or restoration. Accordingly, the circular trajectory emerges only when the input includes functionally distinct neuronal subpopulations that reflect antagonistic interactions. Restricting the analysis to homogeneous neurons eliminates this nonlinear cyclicity, indicating that reciprocal dynamics between distinct functional subgroups are necessary to generate the manifold (Figure S6). The trajectory captures how internal circuit feedback organizes the transition from evoked depression to homeostatic recovery. Thus, the manifold is not merely a low-dimensional projection of BP changes, but a structured expression of the intrinsic feedback dynamics within the IML circuit that generate and regulate these state transitions.

Because such feedback logic is expressed through how population activity evolves over time, we characterized it using a trajectory-based manifold representation rather than a set of instantaneous features. Various strategies exist for characterizing high-dimensional neural activity, including spectral parameterization of periodic and aperiodic components and functional network analysis.^61^^,^^62^ Such frameworks typically summarize neural activity within a given time window into a small set of scalar features, such as band power or network efficiency. Our study instead adds, on top of these scalar features, the continuous path traced by population activity over time, captured through a low-dimensional neural manifold underlying the state dynamics of the neural population,^63^^,^^64^ thereby preserving the temporal ordering of population activity within a low-dimensional representation, an extension well suited to characterizing the BP reflex.

The circular manifold revealed in the IML does not simply mirror the dynamics of BP transitions, but instead reflects a fundamental computational structure by which autonomic circuits enforce homeostasis. BP regulation exemplifies a recurrent process: transient deviations induced by stimulation are followed by a gradual return to baseline. In parallel, population-level neural activity also returns to its original rhythm, and this coordinated trajectory is encoded in a circular manifold that loops back to its origin—representing the homeostatic set point.

While the manifold provides a low-dimensional summary of BP and temporal dynamics, it was not used as a direct predictive input in this study. This decision reflects a conceptual distinction: although the manifold aligns closely with observed physiological transitions, we did not treat it as a mere outcome to be fitted. Rather, we interpreted the manifold as an expression of the recurrent logic that produces both the neural dynamics and the BP responses. In other words, the manifold does not just track homeostasis—it enforces it. To evaluate this embedded logic, we assigned the manifold as the fixed recurrent scaffold in a neural network, allowing synthetic neurons to autonomously generate physiological transitions guided by this structure.

The adopted recurrent neural network is based on FORCE learning, in which the output is recursively fed back into the network, enabling the emergence of self-sustained dynamics that autonomously generate and maintain internal states.^51^ This architecture fundamentally differs from models such as long short-term memory (LSTM) or gated recurrent unit (GRU), where the recurrent connections themselves are updated during training, making it difficult to preserve a stable circuit structure.^65^^,^^66^^,^^67^ In contrast, our model maintains the recurrent architecture defined by the manifold and only updates the output weights, thereby allowing the circuit logic embedded in the manifold to persist while generating time-evolving neural dynamics.^68^^,^^69^

Moreover, rather than treating the manifold as a feature for predicting BP, we interpreted it as a structural scaffold of intrinsic population dynamics and assigned it as the core recurrent architecture of the reservoir. Our primary objective was not to predict BP per se but to model the evolving structure of population neural responses over time from which physiological outputs are derived. By implementing the manifold not as an input feature but as a functional computational structure within the recurrent neural network, the model successfully reproduced both neural and physiological responses—validating the manifold as a reflection of circuit-level internal logic.

The manifold-based analysis and recurrent neural network model proposed here structured the intrinsic dynamics of the IML circuit and implemented them in a predictable manner, thereby increasing both the interpretability and controllability of neural circuits. Although the model was trained using individual neural trajectories from each animal and did not establish a generalized reservoir architecture, it provided a foundation for cross-subject comparison and interpretation through the aligned phase-space structure. One might ask whether such a shared model would remain valid under pathological conditions, where physiological baselines are altered. To address this, we examined animals with chronically elevated BP and found that the same circular manifold structure emerged, indicating that the underlying feedback organization of the circuit persists regardless of disease state. In other words, the manifold reflects not the absolute baseline, but the dynamic logic by which the system restores it. This finding suggests that once a recurrent homeostatic framework is maintained, the manifold-based model can be used to predict physiological transitions even when set points shift. Accordingly, this approach can be extended beyond BP regulation to other autonomic variables such as heart rate, respiration, and body temperature—domains that also rely on circuit-level feedback to maintain homeostasis. By digitizing circuit dynamics around this recurrent structure, the model enables more interpretable and adaptable neuromodulation strategies, potentially informing precision autonomic control, digital twin-based diagnostics, and personalized interventions.

### Limitations of the study

Although the present findings establish a manifold-based framework for reconstructing autonomic state transitions in the spinal IML circuit, several limitations should be acknowledged. First, only male Sprague-Dawley rats were used, and the influence of sex on the circuit-level antagonistic dynamics and the resulting manifold structure was not examined; sympathetic tone is known to differ by sex hormone status, so extending the framework to female animals will be necessary to establish generalizability across sexes. Second, the recurrent neural network reservoir was built from a manifold derived individually for each animal rather than a shared architecture pooled across subjects. Simple linear transformations restricted to translation, rotation, and scaling were enough to align these individually derived manifolds into a common circular structure that shared the same phase relationship with BP across all seven animals. While this shared structure points toward an architecture usable across animals, translating it into a single reservoir that generalizes to a new subject without per-animal calibration remains to be developed.

## Resource availability

### Lead contact

Requests for further information and resources should be directed to and will be fulfilled by the lead contact, Sungmin Park (sungminpark@postech.ac.kr).

### Materials availability

This study did not generate new, unique reagents.

### Data and code availability

• All data reported in this paper will be shared by the lead contact upon request.
• Original code required to reproduce the main findings of this study has been deposited at Zenodo: (https://doi.org/10.5281/zenodo.23409705) and GitHub (https://github.com/Choo-Minhye/spinal-manifold-digital-twin) and is publicly available as of the date of publication.
• Any additional information required to reanalyze the data reported in this paper is available from the lead contact upon request.

## Acknowledgments

This research was supported by the Pioneer Research Center Program through the National Research Foundation of Korea (NRF) funded by the Ministry of Science, ICT & Future Planning (2022M3C1A3081294); the NRF grants funded by the Korea government (MSIT) (RS-2025-00517742) and (MSIT and MOE) (No. RS-2025-17492968); the Basic Science Research Program through the NRF funded by the Ministry of Education (RS-2025-25426604); and the ANCHOR program through the Gyeongbuk ANCHOR CENTER, funded by the Ministry of Education (MOE) and Gyeongsangbuk-do, Republic of Korea (2026-ANCHOR-15-118).

## Author contributions

Conceptualization, M. Choo and M. Cho; methodology, M. Choo and M. Cho; investigation, M. Choo, M. Cho, C.O., and S.H.; software, M. Choo; formal analysis, M. Choo; writing – original draft, M. Choo, C.H., Y.-M.S., and S.P.; writing – review and editing, M. Choo, C.H., Y.-M.S., and S.P.; resources, J.L.; supervision, S.P.; funding acquisition, S.P.

## Declaration of interests

The authors declare no competing interests.

## STAR★Methods

### Key resources table

**Table:** 

REAGENT or RESOURCE	SOURCE	IDENTIFIER
**Chemicals, peptides, and recombinant proteins**		

Urethane	Sigma-Aldrich	U2500
Heparinized saline	Sigma-Aldrich	H5515

**Experimental models: Organisms/strains**		

Rat: Sprague-Dawley, male, 8–10 weeks	Orient Bio, Korea	N/A

**Software and algorithms**		

Klusta	Rossant et al.^70^	https://github.com/kwikteam/klusta
Phy	Rossant et al.^70^	https://github.com/cortex-lab/phy
MATLAB	Math Works	https://www.mathworks.com
OpenEx	Tucker-Davis Technologies	https://www.tdt.com
Scikit-learn (Isomap, PCA, KMeans, LinearRegression, MinMaxScaler, mutual_info_regression)	Pedregosa et al.^71^	https://scikit-learn.org/stable/
PyTorch	Paszke et al.^72^	https://pytorch.org
ripser.py	Tralie et al.^73^	https://github.com/scikit-tda/ripser.py
dcor	Ramos-Carreño et al.^74^	https://github.com/vnmabus/dcor

**Deposited data**		

Original code	This paper	Zenodo: https://doi.org/10.5281/zenodo.23409705
**Other**		

16-channel silicon probe (A1x16-Poly2s-5mm-50s-177-A16)	NeuroNexus Technologies	A1x16-Poly2s-5mm
Pressure transducer (BP-102)	iWorx	BP-102
Data acquisition system (IX-RA-834)	iWorx	IX-RA-834
Waveform generator (IZ2-32)	Tucker-Davis Technologies	IZ2-32

### Experimental model and study participant details

#### Animals

Adult male Sprague-Dawley rats (age: 8–10 weeks; weight: 270–320 g) were obtained from Orient Bio (Rep. of Korea) and Koatech (Rep. of Korea). Animals were housed under a 12 h light/dark cycle with *ad libitum* access to food and water. All procedures were approved by the POSTECH Institutional Animal Care and Use Committee (POSTECH-2023-0094 and POSTECH-2024-0084) and conducted in accordance with the NIH Guide for the Care and Use of Laboratory Animals. Only male rats were used in this study; the influence of sex on the results was not examined and represents a limitation of the study’s generalizability.

### Method details

#### Study approval and clinical samples

All surgical and experimental protocols were approved by the Institutional Animal Care and Use Committee at Pohang University of Science and Technology (POSTECH-IACUC; approval numbers: POSTECH-2023-0094 and POSTECH-2024-0084) and followed the animal experiment guidelines from the NIH Guide for the Care and Use of Laboratory Animals. We used the adult male Sprague-Dawley rats (age: 8–10 weeks; weight: 270–320 g; provided by Orient Bio, Rep. of Korea, colony reference at Charles River Laboratories, and by Koatech, Rep. of Korea, colony reference at Envigo). We performed surgeries with the rat subjects for non-survival acute experiments, and thus, at the end of the experiments, the subjects were euthanized using CO2 gas.

#### Anesthesia and surgical procedures

Rats were initially anesthetized with 5% isoflurane in oxygen (0.4–0.6 L/min flow) for 5–10 min to induce loss of consciousness. Subsequently, a urethane solution (1.2–1.4 g/kg, 0.2 g/mL in saline; Sigma–Aldrich, U2500) was administered intraperitoneally to maintain deep anesthesia throughout the experiment. Additional doses (0.3–0.6 g/kg) were delivered as needed based on reflex testing. If spontaneous respiration was unstable, a tracheal intubation was performed to secure the airway and ensure adequate ventilation.

To monitor arterial blood pressure, a polyethylene catheter (SP-10; Natsume Seisakusho Co.) was inserted into the left femoral artery. Animals were then positioned on a stereotaxic frame (RWD Life Science, model 68002), and a laminectomy was performed to expose the spinal cord at vertebral levels T11–T12. For stabilization during neural recordings and stimulation, custom clamps were applied to the transverse processes of T10 and T13 vertebrae.

Electrical stimulation and neural recordings were targeted to the spinal cord segments corresponding to the T13–L1 region. Two polymide-coated stainless steel wires (150 μm diameter; GoodFellow), with only the tips deinsulated, were inserted 1.0–1.2 mm deep into the ventral spinal cord on the left side at the T13 level for bipolar stimulation. For neural recordings, reference and ground electrodes (200 μm stainless steel) were inserted on the right side at T13. A 16-channel silicon probe (A1x16-Poly2s-5mm-50s-177-A16; NeuroNexus Technologies) was implanted into the left ventral spinal cord spanning the T13–L1 region, with insertion depth adjusted within 300 μm to optimize multi-unit signal detection. All procedures were conducted under stable anesthetic depth and physiological condition.

#### Signal acquisition and preprocessing

Neural signals were recorded and electrical stimuli were delivered using an integrated data acquisition and stimulation system (Tucker-Davis Technologies, TDT). Extracellular potentials were digitized at 24,414 Hz using a headstage (ZC16), a digitizer (PZ5-32), and a headstage adapter (ZCA-DIP16). The system was controlled via a real-time processor (RZ5), and all connections were electrically isolated and shielded within a custom-built Faraday cage (copper mesh and aluminum frame). A high-pass filter (>0.5 Hz) and 60 Hz notch filter were applied online using OpenEx software to reduce baseline drift and mains interference. Neural recordings were stored in TDT tank format and converted to MATLAB (∗.mat) files using the TDT MATLAB SDK for further processing.

Electrical stimulation was applied via a bipolar electrode inserted into the spinal cord, using a programmable waveform generator (IZ2-32, TDT), which was isolated from ground using a battery module (LM48M-250, TDT) to prevent feedback and noise coupling into the neural recordings. Stimulation parameters (waveform, duration, and frequency) were defined in real time through the same OpenEx software suite, and stimulation onset/offset times were logged for synchronization with physiological recordings. Stimulation frequency (40 Hz) was selected based on preliminary comparisons of neural manifold formation and blood pressure responses across multiple stimulation frequencies (Figure S7).

Arterial blood pressure was continuously measured via a femoral artery catheter connected to a pressure transducer (BP-102, iWorx) and digitized at 100 Hz using an acquisition system (IX-RA-834, iWorx). The tubing was routed outside the Faraday cage to minimize electrical interference. The catheter was flushed regularly with heparinized saline (100 IU/mL, Sigma-Aldrich H5515) to prevent clot formation. All blood pressure recordings were exported as ∗.mat files for offline analysis. From the raw trace, mean arterial pressure (MAP) was estimated via low-pass filtering (<0.5 Hz).

#### Single-unit spike sorting and classification of IML neurons

Neural signals recorded from the IML were analyzed to extract single-neuron activity patterns. Spike sorting was performed using the open-source Klusta suite, which automatically detects spike waveforms from high-pass filtered data (300–6000 Hz) and groups them into clusters representing candidate single neurons. These clusters were then manually reviewed and refined using the Phy graphical interface, which provides key quality metrics such as spike waveform shape and autocorrelograms. Clusters were excluded if they exhibited a refractory period shorter than 2 ms, lacked a clear V-shaped waveform, or appeared across more than 10 channels—criteria suggestive of noise or multi-unit contamination.

To identify neurons with consistent responses to stimulation, we computed the phase-locking value (PLV) using the instantaneous firing rate of each neuron across repeated trials.

The PLV was defined as:

(Equation 1) $$PLV=\left|\sum_{k}\frac{1}{N}e^{j\left(x_{k}-x_{ref}\right)}\right|$$

where *N* is the number of time points,

*x*_k_ is the firing rate at time *k*, and *x*_r*ef*_ is a cosine reference signal phase-matched to the stimulation cycle. Neurons with PLV >0.1 were classified as responsive, indicating stable and phase-consistent modulation by stimulation. Among the recorded IML neurons, we classified their response types based on changes in firing rate during stimulation. Neurons that exhibited a consistent increase in firing rate were categorized as *activated*, reflecting excitatory modulation by stimulation input. In contrast, neurons showing a reliable decrease in firing rate were labeled as *suppressed*, suggesting inhibition during the stimulus period. Neurons that did not show any significant or consistent change in firing rate across repeated trials were categorized as *non-responsive* and were excluded from downstream analyses. This classification enabled subsequent investigations into how functionally distinct IML neuronal subtypes contribute to the population dynamics evoked by stimulation.

#### Extraction of latent dynamics from IML population activity

To uncover the low-dimensional dynamics embedded in IML population activity, we applied nonlinear dimensionality reduction to time-varying firing patterns of single neurons. Raw spike trains were first converted into smoothed firing rates by binning spikes in sliding windows (0.2–0.6 s, empirically chosen per animal) and convolving with a Gaussian kernel (window: 3 s, σ = time step). These vectors defined the high-dimensional neural state space, where each axis corresponded to the firing activity of one neuron.

Due to noise and redundancy in high-dimensional spaces, direct interpretation of population dynamics can be difficult. We used Isomap (Scikit-learn implementation), a nonlinear embedding technique that preserves geodesic distances between neural states, to obtain a reduced set of latent coordinates.

Isomap constructs a neighbor graph in the high-dimensional firing-rate space by connecting each neural state *x*_*i*_ to its *k* nearest neighbors under Euclidean distance. Geodesic distances between all pairs of states are then approximated as shortest-path distances on this graph:

(Equation 2) $$D_{ij}^{geo}=\min_{path\;p:i\to j}\sum_{\left(k,l\right)\in p}\left|x_{k}-x_{l}\right|$$

Classical multidimensional scaling is then applied to the resulting geodesic distance matrix to obtain a low-dimensional embedding $z_{i}\in R^{d}$ that best preserves these pairwise distances, by minimizing the stress function $\Sigma_{i,j}{\left(D_{\text{ij}}^{\text{geo}}-\left|\left|z_{i}-z_{j}\right|\right|\right)}^{2}\text{.}$

This approach has been shown to better capture smooth neural trajectories than linear alternatives like principal component analysis.^75^ We evaluated this rationale directly by comparing Isomap against PCA, LLE, and an autoencoder, as described below. The resulting low-dimensional manifold retained key temporal features of population activity, while suppressing spurious variability, enabling clearer interpretation of circuit-level transitions. Each latent axis reflected coordinated changes in neuronal firing across the IML, offering a compact representation of collective neural dynamics.

#### Comparison with alternative dimensionality reduction methods

To evaluate whether Isomap uniquely preserves the geometric structure of IML population activity, we compared it with three alternative dimensionality reduction methods spanning linear and nonlinear approaches: principal component analysis (PCA), locally linear embedding (LLE), and an autoencoder, all implemented with 6 latent dimensions to match the dimensionality used for Isomap. LLE was implemented using Scikit-learn with 5 nearest neighbors and the standard reconstruction method. The autoencoder consisted of a single hidden layer (32 units) with ReLU activation in both the encoder and decoder, trained in PyTorch using the Adam optimizer to minimize mean squared reconstruction error over 1000 epochs, with a fixed random seed for reproducibility.

(Equation 3) $$L_{AE}=\frac{1}{N}\sum_{i=1}^{N}{\left|\left|x_{i}-{\hat{x}}_{i}\right|\right|}^{2}$$

Where *x*_*i*_ is the *i*-*th* high-dimensional firing-rate vector and ${\hat{x}}_{i}$ is its reconstruction from the 6-dimensional latent code.

To assess whether each method’s latent representation preserved the circular topology observed with Isomap, we computed persistent homology (ripser package, coefficient field ℤ2, maximum homology dimension 2) on each normalized latent point cloud, and quantified the separation between the longest-lived and second-longest-lived first homology (H1) feature.

(Equation 4) $$\Delta H_{1}=\left(d_{1}-b_{1}\right)-\left(d_{2}-b_{2}\right)$$

where (*b*_1_,*d*_1_) and (*b*_2_,*d*_2_) are the birth and death filtration values of the first and second longest-lived H1 features.

To test whether each method preserved the geodesic distance structure of the original high-dimensional firing-rate space, we constructed a k-nearest-neighbor graph (k = 5) from the raw firing-rate vectors, computed pairwise shortest-path distances on this graph as a method-independent geodesic reference.

(Equation 5) $$D_{ij}^{geo}=min_{path\;p:i\to j}\sum_{\left(k,l\right)\in p}\left|\left|x_{k}-x_{l}\right|\right|$$

We then correlated these geodesic distances with the pairwise Euclidean distances between points in each method’s latent space to obtain a geodesic preservation score.

(Equation 6) $$r_{geo}^{2}={\left[corr\left(D_{ij}^{geo},\left|\left|z_{i}-z_{j}\right|\right|\right)\right]}^{2}$$

This score was computed at each latent dimensionality from 1 to 6, where $z_{i}\in R^{d}$ denotes the *d* d-dimensional latent representation of point *i*.

To evaluate the functional relationship between each method’s latent representation and blood pressure, we fit an in-sample linear regression predicting blood pressure from the first two latent dimensions of each method, after min-max normalization of both predictors and target. Statistical comparisons between Isomap and each alternative method were performed using paired Wilcoxon signed-rank tests across *n* = 7 animals.

#### Quantifying latent dimension contributions to hemodynamic state transitions

To assess how each latent axis (DIM1, DIM2) contributes to blood pressure dynamics, we applied a time-resolved ridge regression model using the *RidgeCV* class in Scikit-learn. The Isomap-projected neural state at each time point was defined as a 2D vector *z*_*t*_ = [*z*_1, *t*_*z*_2, *t*_] and blood pressure at time *t* as *y*_*t*_.

The regression model took the form: ${\hat{y}}_{t}=\beta_{1}z_{1,t}+\beta_{2}z_{2,t}$ with a regularization term αΣ $\beta_{i}^{2}$, where α was automatically selected via cross-validation. Ridge regression was performed in a sliding window (5 s window, 1 s step), and the absolute values |*β*_1(*t*)_| and |*β*_2(*t*)_| were taken as time-varying contribution magnitudes of DIM1 and DIM2, respectively.

To capture relative dominance of each dimension, we computed a dominance index: (*t*) = |*β*_1(*t*)_|−|*β*_2(*t*)_|. Positive *D*(*t*) indicate greater contribution from DIM1, and negative *D*(*t*) indicated dominance of DIM2. Time segments were classified based on whether *D*(*t*) was significantly different from zero using a two-tailed paired *t* test across animals, with significance defined as *p* < 0.001 sustained for at least 5 consecutive time steps (∼5 s). These periods were marked as functionally distinct encoding epochs in Figure 3D. To identify neurons contributing to each latent dimension, we calculated the Pearson correlation coefficient *r*_*i*_ between the smoothed firing rate of each neuron and the time-series of each dimension *z*_*d*_(*t*), where d∈(1,2):

(Equation 7) $$r_{i}^{\left(d\right)}=\frac{Cov\left(x_{i}\left(t\right),z_{d}\left(t\right)\right)}{\sigma_{x_{i}},\sigma_{z_{d}}}$$

Neurons with the highest $r_{i}^{\left(1\right)}$, or $r_{i}^{\left(2\right)}$ were labeled as DIM1-or DIM2-selective, respectively (the top five neurons ranked by correlation coefficient). These neurons were visualized in Figure 3E, and further used for subgroup-specific phase alignment analyses.

Lastly, to characterize the evolving relationship between latent dimensions and blood pressure, we performed a sliding-window Pearson correlation between each *z*_*d*_(*t*) and *y*_*t*_, using a 10 s window with 1 s steps, resulting in a time-varying neural-physiological coupling profile.

#### Nonlinear correlation analysis between neural features and blood pressure

To evaluate how well the circular manifold of IML population activity encodes physiological state transitions, compared to individual neuronal firing, we assessed the statistical dependency between blood pressure (ΔBP) and two types of neural features: 1) the latent phase of the neural manifold and 2) the firing rate of individual IML neurons. Given the nonlinear nature of both neural and physiological signals, we adopted two correlation metrics designed to capture general statistical associations: distance correlation (dCor) and the mutual information (MI).

Distance correlation was used to quantify localized temporal associations within each stimulation trial. It was selected because it detects both linear and nonlinear dependencies without assuming monotonicity or directionality. That is, dCor measures the degree of co-variation based on pairwise distances, rather than the sign or shape of the relationship. This makes it well-suited for detecting complex interactions between neural and physiological states. Let $x_{1}\in R^{n}$ be a time series of either latent phase or neuronal firing rate, and $x_{2}\in R^{n}$ be the corresponding blood pressure trace. The distance correlation *D*(*x*_1_,*x*_2_) is defined as:

(Equation 8) $$D\left(x_{1},x_{2}\right)=\frac{V\left(x_{1},x_{2}\right)}{\sqrt{V\left(x_{1},x_{1}\right)V\left(x_{2},x_{2}\right)}}$$

Where *V*(·) denotes the distance variance or covariance. Distance correlation was computed using the dcor python package in each of six temporal bins, allowing comparison between neural features in encoding ΔBP across time.

To assess overall encoding fidelity over the full stimulation period, we additionally computed the mutual information(MI). MI captures nonlinear dependencies by estimating the mutual information between continuous variables using a k-nearest neighbor–based method. It quantifies how much information a neural feature *x* (firing rate or manifold phase) conveys about blood pressure *y*. Formally, mutual information is:

(Equation 9) $$I\left(x;y\right)=\int_{x}\int_{y}p\left(x,y\right)\log\left(\frac{p\left(x,y\right)}{p\left(x\right)p\left(y\right)}\right)dxdy$$

Where *p*(*x*,*y*) is the joint probability distribution, and *p*(*x*), *p*(*y*) are marginal distributions. MI was calculated using the *mutual_info_regression f*unction in scikit-learn. Input features were normalized firing rates or latent phases; the target was either interpolated blood pressure or physiological latency. This allowed direct comparison of the total nonlinear dependence strength between features.

Together, these analyses provided a rigorous basis for comparing the encoding power of latent dynamics and individual neuron activity in representing autonomic state transitions.

#### Geometric normalization of the latent manifold via linear transformation

To enable direct comparison of latent neural manifolds across subjects and trials, we applied a sequence of linear geometric transformations that preserve the internal structure of the manifold while aligning them to a common reference frame. The goal was to maintain the essential dynamics and shape of each trajectory while minimizing deformation and standardizing their position along a shared physiological axis defined by blood pressure (ΔBP). All manifolds were first projected into a two-dimensional latent space $z\left(t\right)\in R^{2}$, and then transformed to ensure consistent temporal evolution and physiological alignment. Because some trajectories exhibited counterclockwise progression—indicative of time flowing in the opposite direction—we applied a reflection to unify the directionality of all manifolds. Specifically, a vertical flip matrix *F* as used to reverse the sign of the second latent dimension:

(Equation 10) $$z^{\prime \prime }\left(t\right)=F\cdot Z\left(t\right),where\;F=\left[\begin{array}{cc} 1 & 0\\ 0 & -1 \end{array}\right]$$

Next, we rotated the reflected manifold such that the point corresponding to the maximum ΔBP value was aligned with the positive Y axis. This step ensured that the Y-dimension of the resulting trajectory would serve as a physiological encoding axis across subjects: *z*′(*t*) = *R*·*z*″(*t*) where *R* denotes the rotation matrix computed to place the ΔBP peak vertically. To allow shape-preserving comparisons across animals with differing absolute ΔBP ranges, we then normalized the rotated manifolds onto a unit circle by isotropic scaling: *z*_*final*_(*t*) = *S*·*z*′(*t*). Here, S is the scaling factor that adjusts the maximum radial distance from the origin to unity. This final transformation ensured that all latent trajectories were aligned to a common ΔBP-oriented frame and shared the same circular domain, allowing for consistent evaluation of physiological encoding without losing intrinsic geometric features.

#### Recurrent neural network model with manifold driven FORCE learning

We implemented a recurrent neural network (RNN) model using the FORCE learning algorithm to decode both physiological (blood pressure) and neural population responses from latent manifold features. The latent input $X\left(t\right)\in R^{2}$ (two-dimensional) was derived from Isomap projection of population spike activity and served as input to a recurrent reservoir with units. The reservoir dynamics followed the standard formulation:

(Equation 11) $$r\frac{dx\left(t\right)}{dt}=-x\left(t\right)+gJr\left(t\right)+W_{z}X\left(t\right)$$

where $x\left(t\right)\in R^{N}$ is the membrane potential, $r\left(t\right)=\tanh\left(x\left(t\right)\right)\in R^{N}$ is the nonlinear activation, $J\in R^{NxN}$ is the fixed recurrent connectivity matrix initialized with sparse Gaussian weights, and $W_{z}\in R^{Nx2}$ is the input weight matrix. The gain factor **g** controlled the degree of recurrent chaos.

The readout from the reservoir was computed as a linear combination of the reservoir activity: ${\hat{y}}_{t}=W_{r}^{T}r\left(t\right)$ for blood pressure prediction, and $\hat{Z}\left(t\right)=W_{n}^{T}r\left(t\right)$ for neural response reconstruction, where $W_{r}\in R^{N}$ and $W_{n}\in R^{NxD}$ are the respective output weight matrices.

During training, only the output weights W_*r*_ and W_*n*_ were updated using recursive least squares (RLS) according to the FORCE learning rule. The output error was defined as $e\left(t\right)=\hat{Y}\left(t\right)-Y\left(t\right)$, and the output weights were adaptively adjusted at each timestep according to the FORCE learning rule:

(Equation 12) $$W_{r}\left(t+1\right)=W_{r}\left(t\right)-P\left(t\right)r\left(t\right)e\left(t\right)$$

(Equation 13) $$P\left(t+1\right)=P\left(t\right)-\frac{P\left(t\right)r\left(t\right)r{\left(t\right)}^{T}P\left(t\right)}{1+r{\left(t\right)}^{T}P\left(t\right)r\left(t\right)}$$

Where $P\left(t\right)\in R^{NxN}$ is the inverse correlation matrix. An analogous rule was used to update W_*n*_ for neural response reconstruction. The reservoir connectivity *J* and input projection W_*z*_ remained fixed throughout training.

By maintaining the recurrent structure defined by the manifold-derived latent space and adapting only the output weights, the model preserved the circuit-level organization while generating dynamic, temporally-evolving neural activity and decoding target signals.

Hyperparameters for the FORCE learning model (τ = 0.1, dt = 0.1, g = 0, initial P = I/5) followed the standard formulation of Sussillo & Abbott (2009).^51^ The number of time steps (T = 240) was determined directly by the experimental recording duration (30 s pre-stimulation +90 s post-stimulation, sampled at 0.5 s intervals), and the reservoir size (*N*_*res*_ = 200) was set to be approximately proportional to the mean number of neurons recorded per animal (30–50 units), consistent with typical reservoir-to-input scaling in reservoir computing applications. The number of training iterations (itr = 100) was empirically confirmed to be sufficient for readout weight convergence in our dataset. For the Isomap embedding, k = 5 nearest neighbors and d = 6 latent dimensions were adopted following standard practice for datasets of this scale. Smoothing window sizes (0.2–0.6 s) were chosen empirically per animal to balance noise suppression with temporal resolution.

#### Computational complexity

The computational cost of the proposed framework is dominated by two stages. Isomap embedding scales as *O*(*N*^2^*logN*) with the number of neurons *N*, due to construction of the neighbor graph and computation of pairwise geodesic distances. FORCE learning of the recurrent reservoir scales as $O\left(itr\times T\times N_{res}^{2}\right)$, where *T* is the number of time steps, itr is the number of training iterations, and *N*_*res*_ is the number of reservoir units, reflecting the recursive least-squares update of the *N*_*res*_ × *N*_*res*_ inverse correlation matrix *P*(*t*) at each time step. In our implementation (*N*_*res*_ = 200, T = 240, itr = 100), training completed in 5.81 s on a single Intel CPU core, with no GPU acceleration required.

### Quantification and statistical analysis

Statistical analyses were performed in Python (SciPy, scikit-learn); MATLAB was used only for spike sorting and was not used for any statistical testing. Throughout the manuscript, n refers to the number of animals (*n* = 7) unless a figure legend or the text states otherwise, significance is denoted as ∗*p* < 0.05, ∗∗*p* < 0.01, ∗∗∗*p* < 0.001, and error bars represent the standard error of the mean (SEM) unless otherwise noted.

Pairwise neuron-to-neuron correlation coefficients from recorded spike trains were compared against a time-shuffled control using a two-tailed paired *t* test across *n* = 7 animals (Figure 2D).

Persistent homology was computed on normalized latent point clouds using the ripser package (coefficient field ℤ2, maximum homology dimension 2). Topological significance of the circular manifold was assessed by the separation between the longest-lived and second-longest-lived first homology (H1) feature (ΔH1); this analysis was descriptive and performed on a single representative trial (Figure 3A; Figure S1).

Geodesic distance preservation across Isomap, PCA, LLE, and an autoencoder was evaluated by correlating each method’s embedded Euclidean distances with a method-independent geodesic reference (k-nearest-neighbor graph, k = 5, on the raw firing-rate vectors), computed at latent dimensionalities 1 through 6. Statistical comparisons between Isomap and each alternative method were performed using paired Wilcoxon signed-rank tests across *n* = 7 animals (Figure S3).

The relative contribution of latent dimensions Dim1 and Dim2 to blood pressure was assessed using a time-resolved ridge regression (RidgeCV, scikit-learn; 5 s sliding window, 1 s step; regularization strength selected by cross-validation). A dominance index, D(t), was tested against zero using a two-tailed paired *t* test across *n* = 7 animals; time segments were classified as functionally distinct encoding epochs when D(t) differed significantly from zero (*p* < 0.001) for at least 5 consecutive time steps (∼5 s) (Figure 3D).

Statistical dependency between blood pressure and either the manifold’s latent phase or individual neuronal firing rates was assessed using distance correlation (dcor Python package), computed within each of six temporal bins, and mutual information (mutual_info_regression, scikit-learn), computed over the full stimulation period (Figure 4C).

Manifold-based and single-neuron-based decoding of blood pressure (R^2^, MAE, and MI) were compared across *n* = 7 animals using a two-tailed paired *t* test (Figure 4E).

In-sample linear regression of blood pressure from each method’s first two latent dimensions (Isomap, PCA, LLE, autoencoder) was compared using paired Wilcoxon signed-rank tests across *n* = 7 animals (Figure S3).

Blood pressure prediction accuracy and mean squared error across models (neural dynamic model, linear regression, and convolutional neural network) were compared using two-tailed paired t-tests (neural dynamic model vs. linear regression; neural dynamic model vs. convolutional neural network) across *n* = 7 animals (Figure 6D).

Cluster-wise stimulus responsiveness was assessed by comparing each neuron’s mean firing rate in a pre-stimulation window against a stimulation window using a two-tailed paired *t* test within each response cluster. Clusters 0 and 2 showed significant modulation (*p* = 2.28 × 10^−5^ and *p* = 3.36 × 10^−4^, respectively), Cluster 3 did not reach significance (*p* = 0.290), and Cluster 1, comprising a single neuron, could not be tested (Figure 6I).
