# Supplementary material for: Recurrent latent dynamics in spinal circuits enable a digital twin of autonomic state transitions
Source: iScience. 2026 Oct 26;29(11):117971. doi: 10.1016/j.isci.2026.117971 (PMC13734804; doi:10.1016/j.isci.2026.117971)
Supplement: Supplementary file 1 [file mmc1.pdf]

iScience, Volume *29*

Supplemental information

Recurrent latent dynamics in spinal circuits enable a digital twin of autonomic state transitions

Minhye Choo, Mingeun Cho, Jiho Lee, Changhwa Oh, Sungjun Hong, Chaewon Heo, Young-Min Shon, and Sungmin Park

Supplemental methods

SUPPLEMENTARY ALGORITHM

Manifold-Driven FORCE Learning for Physiological State Decoding

```
Input:
    X_spike   — spike trains from n_neurons IML neurons, T time points
    Y         — blood pressure trace ∈ R^T
    k         — number of nearest neighbors for Isomap
    N_res     — number of reservoir units
    itr, τ, dt — training iterations, time constant, step size

Output:
    W_r  — learned readout weights (blood pressure decoder)
    W_n  — learned readout weights (neural response decoder)

─────────────────────────────────────────────────────────────
Stage 1 — Preprocessing
─────────────────────────────────────────────────────────────

1.  for each neuron i:
            bin spike train in sliding window (0.2–0.6 s, per animal)
            convolve with Gaussian kernel (window 3 s, σ = dt)
            → smoothed firing rate x_i(t)
2.  Stack into X_fr ∈ R^(n_neurons × T)

─────────────────────────────────────────────────────────────
Stage 2 — Latent Manifold Extraction (Isomap)
─────────────────────────────────────────────────────────────

3.  Build k-nearest-neighbor graph G on X_fr (Euclidean distance)
4.  for all pairs (i, j):
            D_ij^geo = min_{path p: i→j}  Σ_{(k,l)∈p}  ||x_k − x_l||
        // shortest-path distance on G  (Eq. 2)
5.  Apply classical MDS to D^geo:
            Find Z = {z_1,...,z_T} ∈ R^2  minimising
            Σ_{i,j} (D_ij^geo − ||z_i − z_j||)^2
            → 2D latent coordinates Z(t) = [z_1(t), z_2(t)]

─────────────────────────────────────────────────────────────
Stage 3 — Geometric Normalization
─────────────────────────────────────────────────────────────

6.  if manifold progresses counterclockwise:
            z''(t) = F · z(t),  F = [[1, 0], [0, −1]]
        // vertical flip to unify trajectory direction  (Eq. 10)
7.  Rotate so that the point of maximum ΔBP aligns with +Y axis:
            z'(t) = R · z''(t)
8.  Scale to unit circle:
            z_final(t) = S · z'(t),  S = 1 / max radial distance

─────────────────────────────────────────────────────────────
```

Stage 4 — FORCE Learning of the Recurrent Reservoir

```
9.  Initialize:
            J  ~ N(0, 1/N_res)    (fixed recurrent connectivity)
            W_z ~ N(0, 1)         (fixed input weights)
            W_r ← 0,  W_n ← 0    (readout weights, to be learned)
            P(0) ← I / α         (inverse correlation matrix)

10. for j = 1 to itr do
    11.     for t = 1 to T−1 do

            // Reservoir state update  (Eq. 11)
        12.         dx = (dt/τ) [−x(t) + g·J·tanh(x(t)) + W_z·z_final(t)]
        13.         x(t+1) ← x(t) + dx
        14.         r(t+1) ← tanh(x(t+1))

            // RLS update of inverse correlation matrix  (Eq. 13)
        15.         P(t+1) ← P(t) − [P(t)·r·r^T·P(t)] / [1 + r^T·P(t)·r]

            // Output error and readout update  (Eq. 12)
        16.         e(t) ← W_r^T·r(t+1) − Y(t+1)
        17.         W_r ← W_r − P(t+1)·r(t+1)·e(t)
                    (analogous update for W_n using neural response target)

    18.     end for
19. end for

20. return W_r, W_n
```

Note: J and W_z remain fixed throughout training. Only W_r and W_n are updated via RLS.

Supplemental Figures

Fig. S1. Two-dimensional neural manifolds consistently capture circular latent structure across animals.

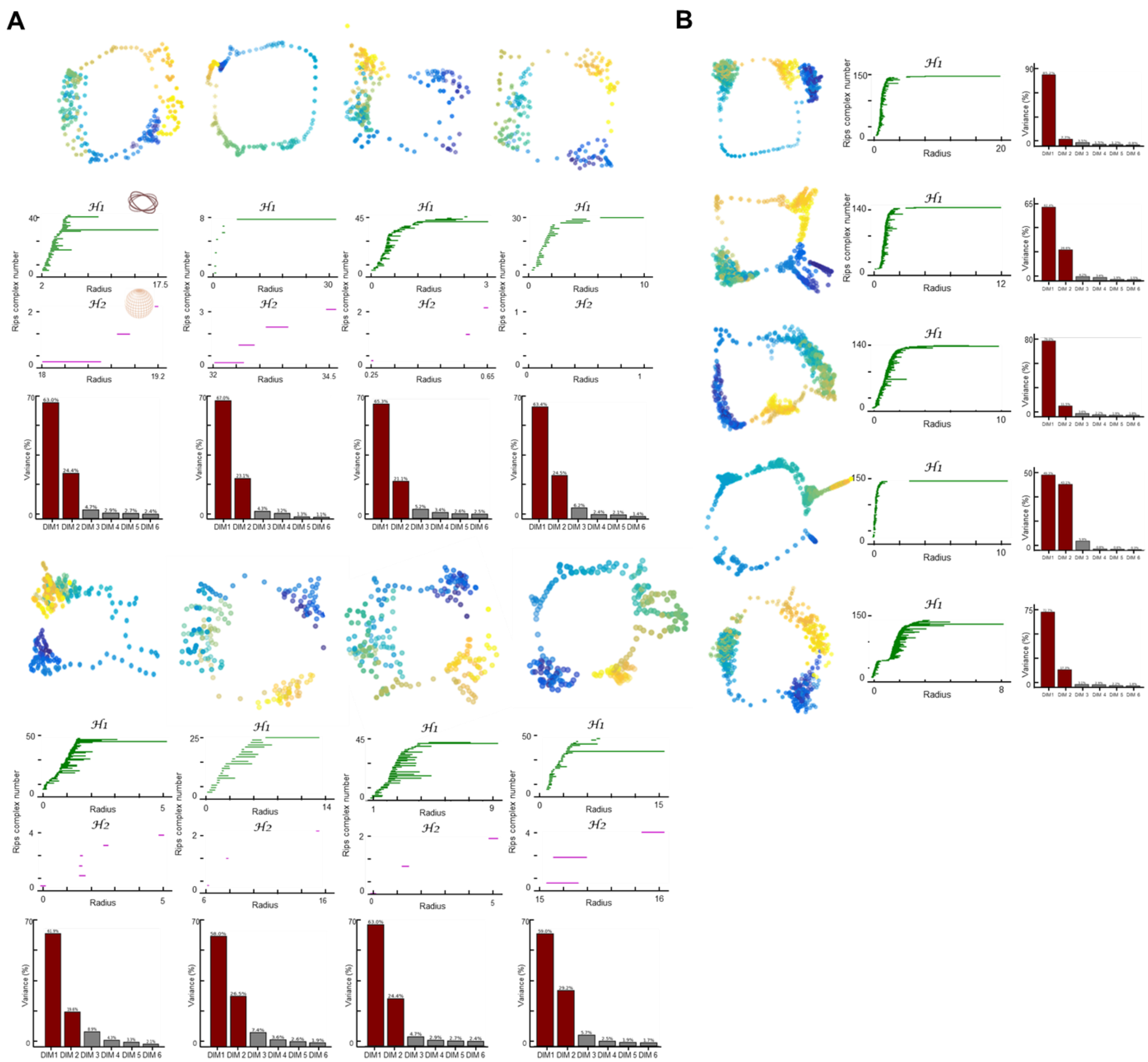

(A) Neural trajectories derived from Isomap embedding of spinal IML activity in control animals (n=8) consistently exhibited circular patterns, with most variance captured by the first two dimensions. Radius–arc length plots and residual variance curves confirm the presence of nonlinear cyclicity and high explanatory power of the leading dimensions.
(B) Similar manifold structures were observed in hypertensive animals (n=5) treated with angiotensin II. Despite physiological perturbation, the first two dimensions accounted for the majority of variance, and circular trajectories were maintained across animals.

Fig. S2. Comparison of Isomap with alternative dimensionality reduction methods in preserving circular manifold geometry and blood pressure encoding.

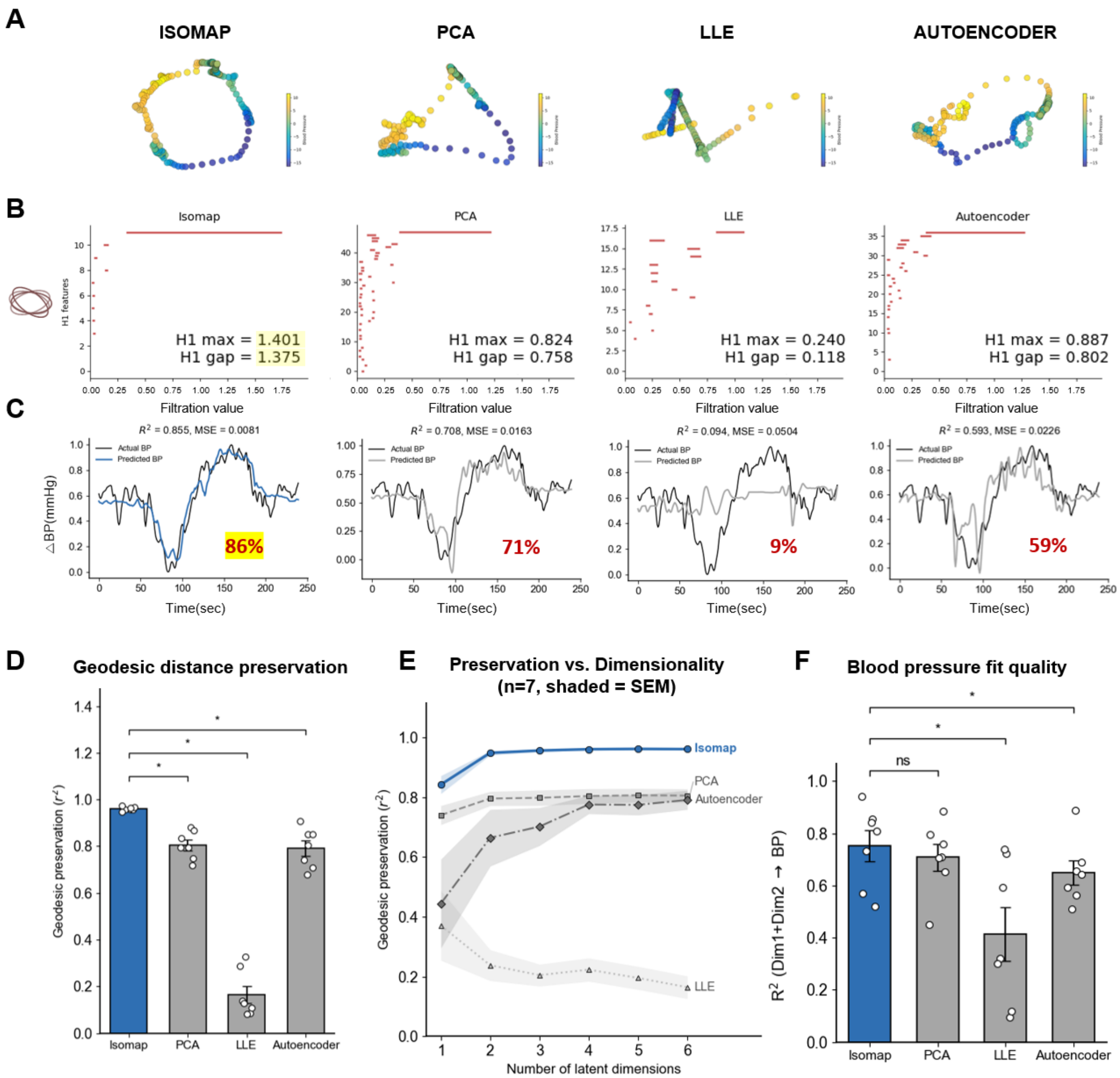

(A) Two-dimensional latent embeddings of IML population activity from a representative trial using Isomap, PCA, LLE, and an autoencoder. Points are colored by simultaneously recorded blood pressure (ΔBP). Only Isomap produced a closed circular trajectory with a smooth gradient of blood pressure values along the trajectory.
(B) Persistent homology diagrams (H1 features) for each embedding method. The H1 gap, defined as the difference in lifetime between the longest-lived and second-longest-lived H1 features, quantifies the presence of a single dominant loop. Isomap yielded the largest H1 gap (1.375), indicating a topologically cleaner circular structure compared to PCA (0.758), LLE (0.118), and the autoencoder (0.802).
(C) Blood pressure prediction from the first two latent dimensions of each method using in-sample linear regression. R^2 values and the percentage of variance explained are shown for each method. Isomap achieved the highest predictive accuracy (R^2 = 0.855, 86%), followed by PCA (R^2 = 0.708, 71%), the autoencoder (R^2 = 0.593, 59%), and LLE (R^2 = 0.094, 9%).
(D) Geodesic distance preservation score (r^2) at two latent dimensions across n = 7 animals. Isomap preserved pairwise geodesic distances significantly better than PCA, LLE, and the autoencoder (paired Wilcoxon signed-rank test, $P < 0.05$). Error bars indicate mean ± SEM.

(E) Geodesic preservation score as a function of the number of latent dimensions (1–6) for each method, averaged across n = 7 animals (shaded region = SEM). Isomap consistently outperformed the other methods across all dimensionalities.
(F) Blood pressure fit quality (R^2 from linear regression on Dim1 + Dim2) across n = 7 animals for each method. Isomap significantly outperformed LLE and the autoencoder ($P < 0.05$), while its performance did not differ significantly from PCA (ns). Error bars indicate mean ± SEM.

Fig. S3. Manifold-based decoding consistently outperforms single-neuron decoding across animals.

A

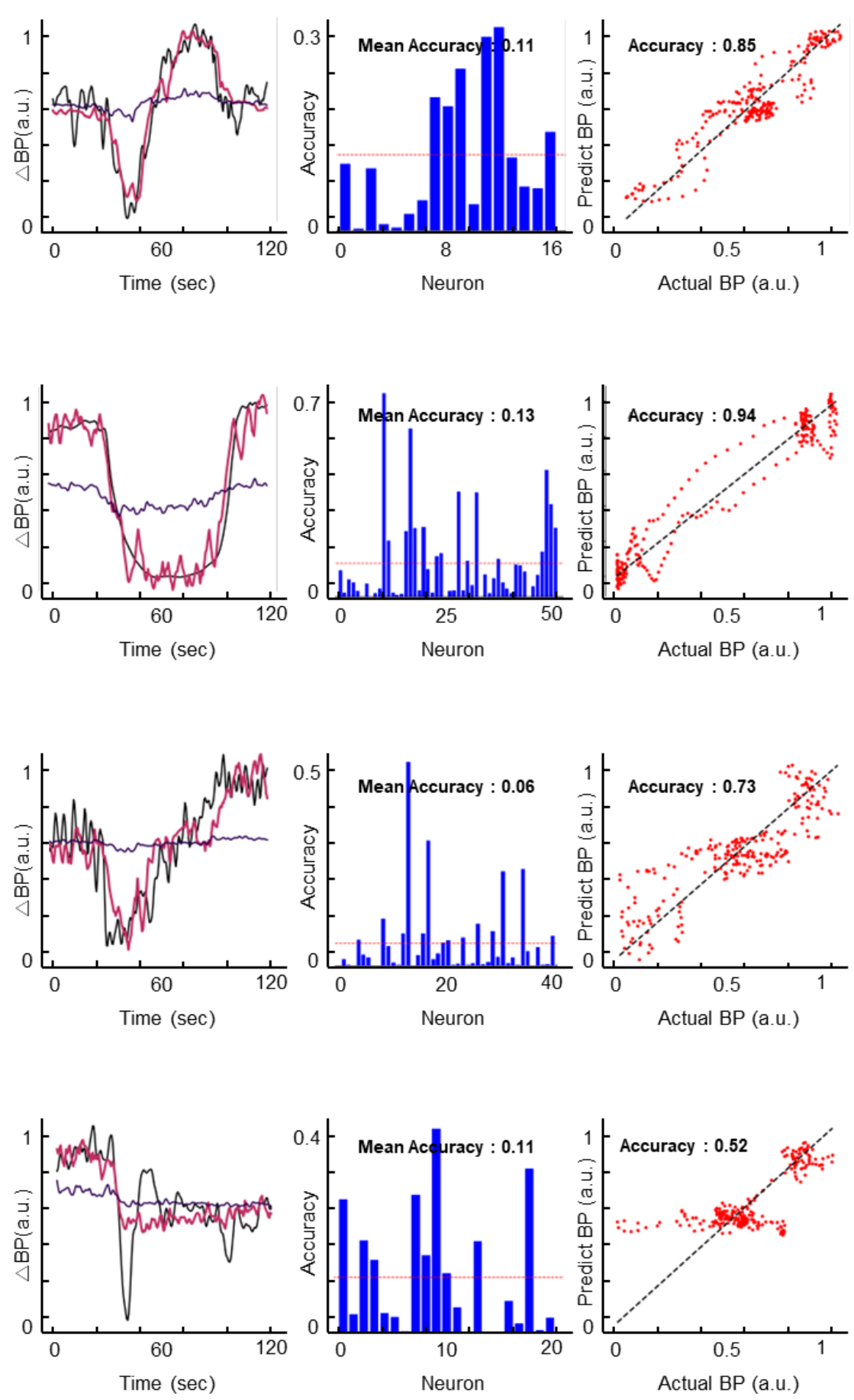

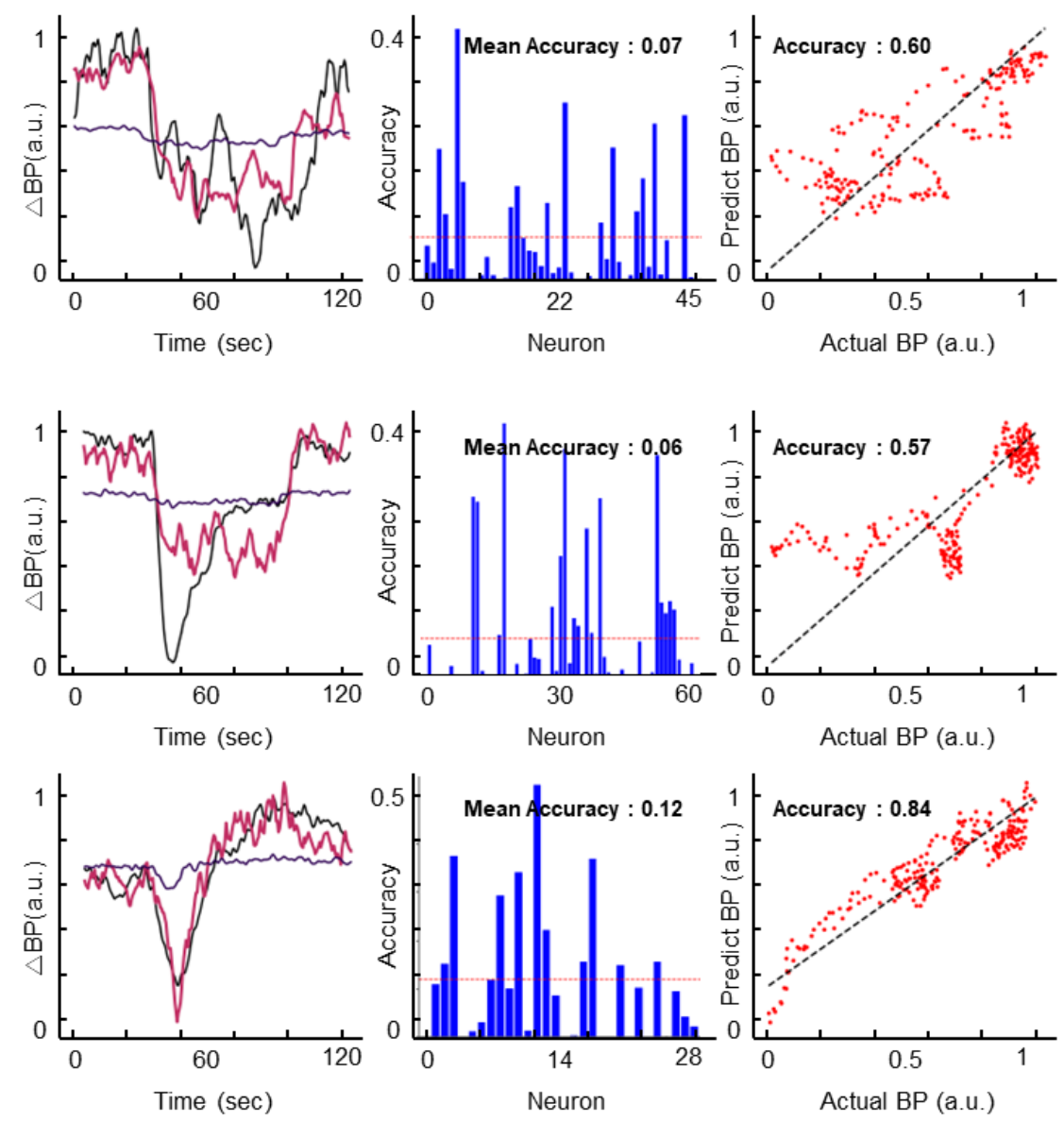

B

	r2_manifold	r2_neuron_avg	mae_manifold	mae_neuron_avg
1	0.94	0.35	0.07	0.35
2	0.85	0.17	0.08	0.17
3	0.57	0.21	0.14	0.21
4	0.60	0.23	0.14	0.23
5	0.84	0.20	0.09	0.20
6	0.52	0.14	0.10	0.14
7	0.73	0.21	0.11	0.21

(A) For each animal (n = 7), blood pressure (ΔBP) was decoded using either the low-dimensional manifold trajectory or individual neuron activities. Left panels, actual (gray) and predicted (magenta) ΔBP traces using the manifold-based model. Middle panels, bar graphs showing the accuracy of each individual neuron in predicting ΔBP, along with the mean accuracy across the population. Right panels, scatter plots comparing actual versus predicted ΔBP using the manifold model; prediction accuracy (R^2) is indicated. **(B)** Summary of decoding performance metrics across animals. The manifold-based model consistently yielded higher R^2 and lower mean absolute error (MAE) compared to the average performance of individual neurons.

These results support the hypothesis that collective neural dynamics captured by the manifold encode blood pressure transitions more robustly than any single neuron.

Fig. S4. Phase-specific encoding of blood pressure transitions by the latent neural manifold.

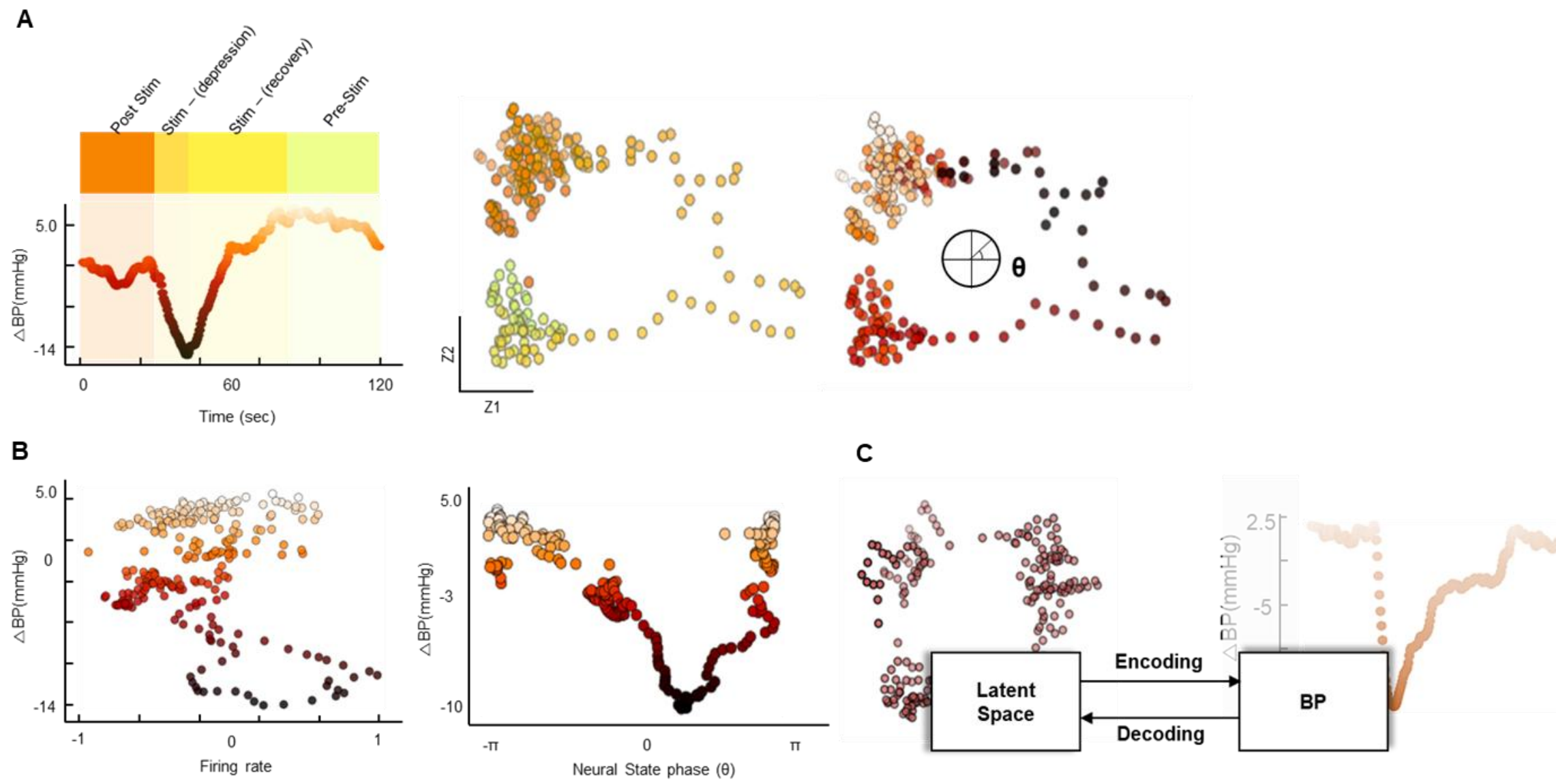

(A) Although identical blood pressure (ΔBP) values appear across different stimulation periods (e.g., early depression vs. recovery), the latent manifold trajectory reveals distinct temporal phases. Angular phase (θ) in the latent space disambiguates these transitions, embedding time and ΔBP jointly in the neural state. **(B)** Aligning individual neuron firing rates with ΔBP fails to uncover structured relationships, whereas aligning ΔBP with the manifold phase reveals a coherent, nonlinear mapping, suggesting that phase more accurately tracks dynamic BP changes than firing rates alone. **(C)** Schematic representation of the bidirectional relationship between the latent space and ΔBP. The manifold supports both encoding of BP from neural activity and decoding of latent features from BP, forming the basis for structured state transition modeling.

Fig. S5. Comparison of Recorded and Model-Generated Neural Manifolds and Physiological Dynamics.

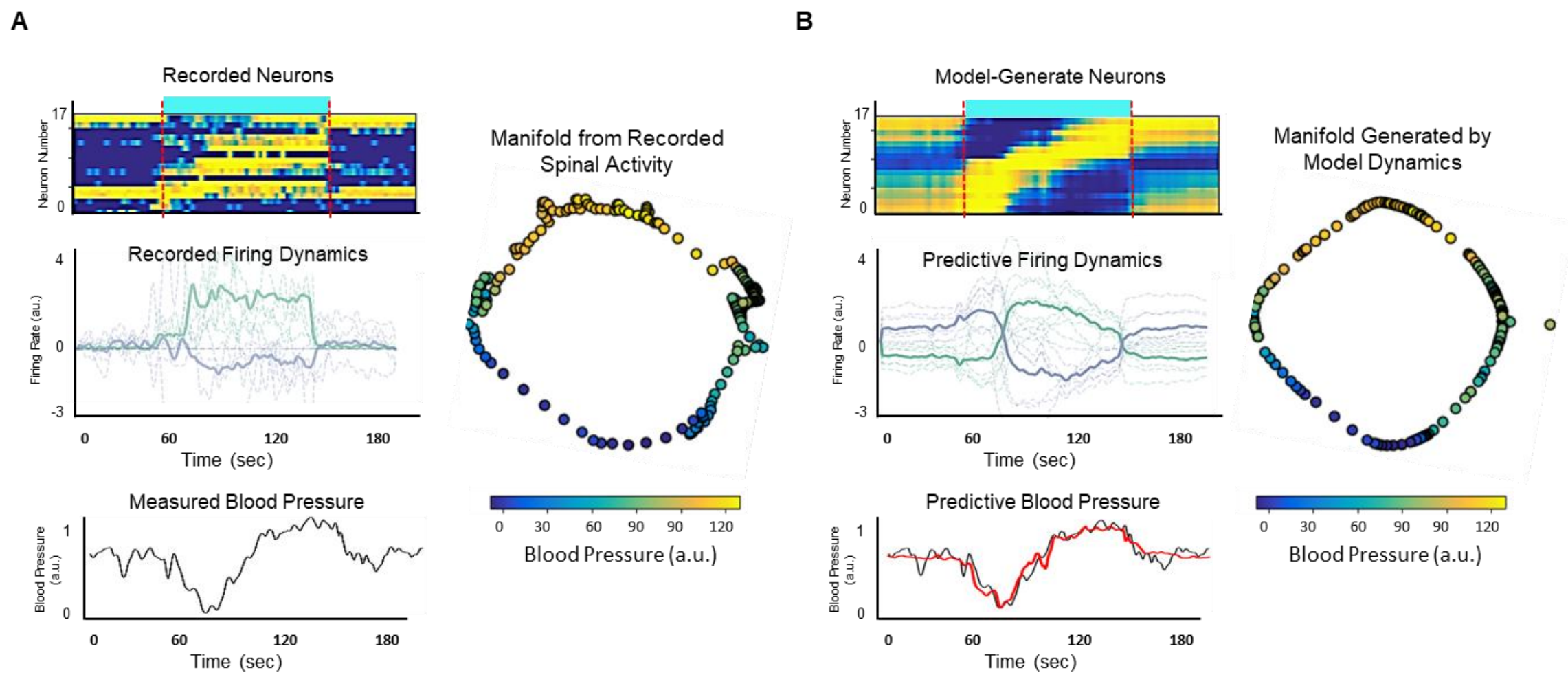

(A) Neural population activity recorded from spinal interneurons during blood pressure modulation. The latent manifold extracted from the recorded firing activity (middle) reflects a cyclic transition tightly coupled to changes in blood pressure (bottom).

(B) Reproduction of the same manifold structure and physiological dynamics using a recurrent neural model. The model-generated firing activity (top) produces a similar cyclic manifold (middle), from which both firing patterns and blood pressure trajectories (bottom) are predicted.

Fig. S6. Circular manifold requires co-existence of functionally distinct neural subpopulations to represent hemodynamic transitions.

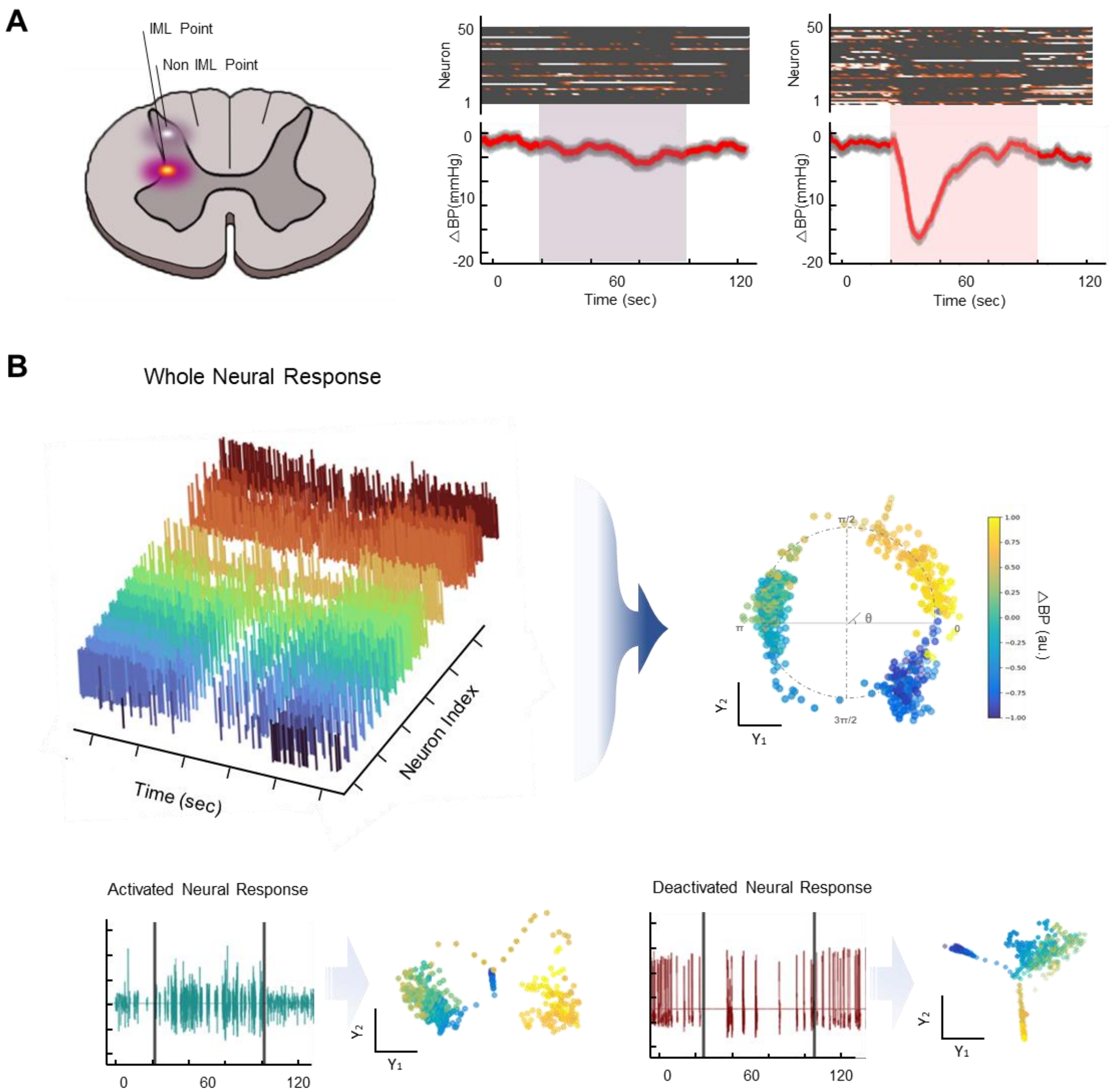

(A) Local stimulation at the intermediolateral (IML) nucleus (left) evoked a clear blood pressure (ΔBP) depression and distinct neural responses (right), whereas stimulation at a non-IML site failed to induce comparable effects, confirming the functional specificity of IML.

(B) Time-aligned neural activity from all recorded neurons (top left) was projected into a low-dimensional manifold (top middle), resulting in a continuous circular trajectory that captured the full ΔBP transition (top right). However, when the manifold was constructed using only neurons that were either activated (bottom left) or deactivated (bottom right), the circular structure was lost. This indicates that the nonlinear cyclicity of the manifold—and its ability to represent transitions between depressed and recovered states—emerges from reciprocal dynamics between antagonistic neural subpopulations.

Fig. S7. Circular manifold requires co-existence of functionally distinct neural subpopulations to represent hemodynamic transitions.

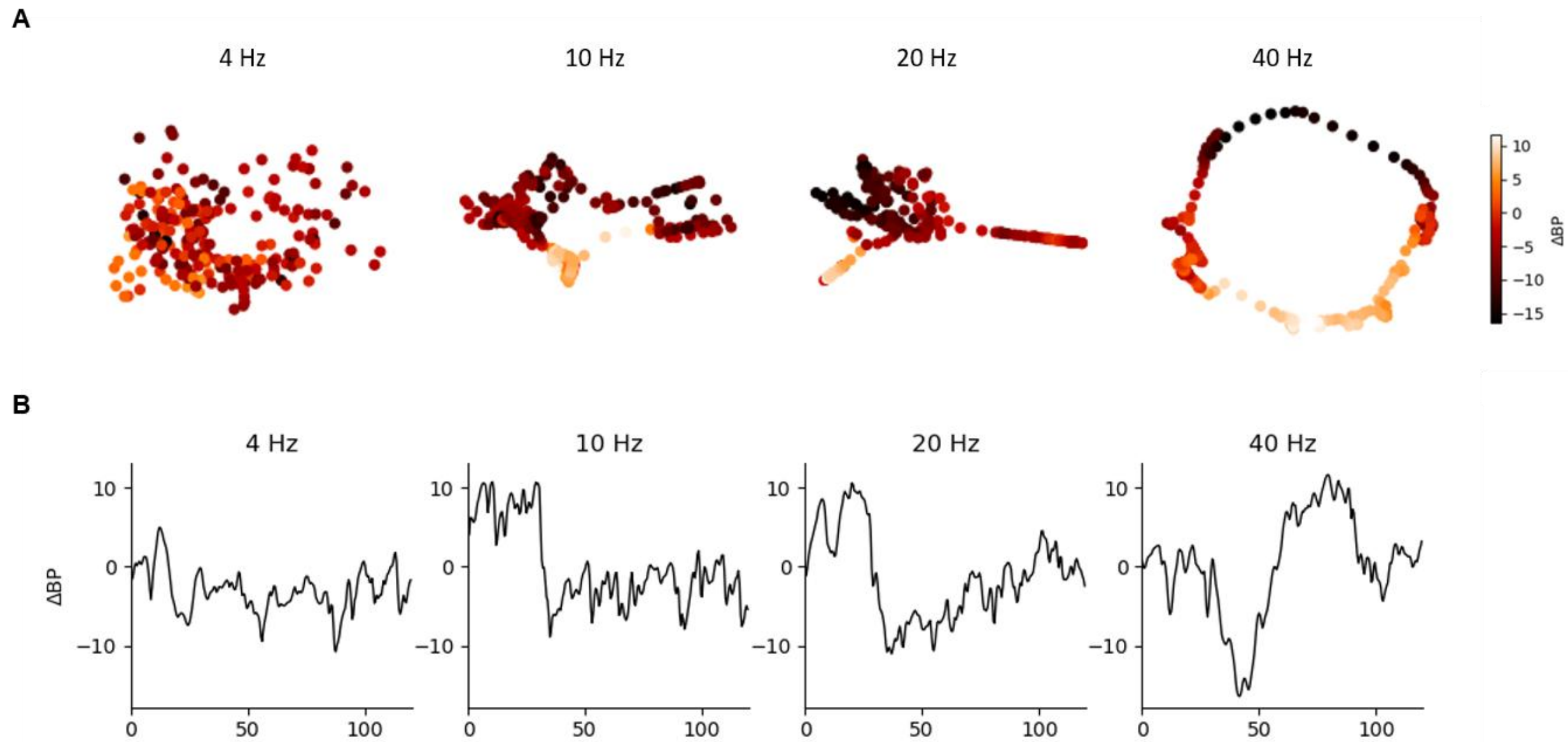

(A) Two-dimensional Isomap projections of IML population activity recorded from the same animal during spinal stimulation at 4, 10, 20, and 40 Hz. Each point represents a single time bin, colored by the simultaneously recorded ΔBP.
(B) Corresponding ΔBP traces for each stimulation frequency shown in (A), aligned to a common 0 to 120 second trial window (30 second pre-stimulation baseline followed by a 90 second post-stimulation period).

Abbreviations: IML, intermediolateral nucleus; BP, blood pressure.

Together, these results demonstrate that the proposed model captures the structured neural dynamics underlying autonomic regulation and enables accurate reconstruction of latent physiological states.
